# A randomized pilot efficacy and safety trial of diazoxide choline controlled-release in patients with Prader-Willi syndrome

**DOI:** 10.1371/journal.pone.0221615

**Published:** 2019-09-23

**Authors:** Virginia Kimonis, Abhilasha Surampalli, Marie Wencel, June-Anne Gold, Neil M. Cowen

**Affiliations:** 1 Division of Genetics and Genomic Medicine, Department of Pediatrics, Univ. of California-Irvine School of Medicine, Orange, California, United States of America; 2 Department of Pediatrics, Loma Linda University Medical School, Loma Linda, California, United States of America; 3 Soleno Therapeutics, Redwood City, California, United States of America; Vanderbilt University, UNITED STATES

## Abstract

**Introduction:**

Prader-Willi syndrome (PWS) is a complex genetic condition characterized by hyperphagia, hypotonia, low muscle mass, excess body fat, developmental delays, intellectual disability, behavioral problems, and growth hormone deficiency. This study evaluated the safety and efficacy of orally administered Diazoxide Choline Controlled-Release Tablets (DCCR) in subjects with PWS.

**Method:**

This was a single-center, Phase II study and included a 10-week Open-Label Treatment Period during which subjects were dose escalated, followed by a 4-week Double-Blind, Placebo-Controlled Treatment Period.

**Results:**

Five female and eight male overweight or obese, adolescent and adult subjects with genetically-confirmed PWS with an average age of 15.5±2.9 years were enrolled in the study. There was a statistically significant reduction in hyperphagia at the end of the Open-Label Treatment Period (-4.32, n = 11, p = 0.006). The onset of effect on hyperphagia was rapid and greater reductions in hyperphagia were seen in subjects with moderate to severe Baseline hyperphagia (-5.50, n = 6, p = 0.03), in subjects treated with the highest dose (-6.25, n = 4, p = 0.08), and in subjects with moderate to severe Baseline hyperphagia treated with the highest dose (-7.83, n = 3, p = 0.09). DCCR treatment resulted in a reduction in the number of subjects displaying aggressive behaviors (-57.1%, n = 10, p = 0.01), clinically-relevant reductions in fat mass (-1.58 kg, n = 11, p = 0.02) and increases in lean body mass (2.26 kg, n = 11, p = 0.003). There was a corresponding decrease in waist circumference, and trends for improvements in lipids and insulin resistance. The most common adverse events were peripheral edema and transient increases in glucose. Many of the adverse events were common medical complications of PWS and diazoxide.

**Conclusion:**

DCCR treatment appears to address various unmet needs associated with PWS, including hyperphagia and aggressive behaviors in this proof-of-concept study. If the results were replicated in a larger scale study, DCCR may be a preferred therapeutic option for patients with PWS.

## Introduction

Prader-Willi syndrome (PWS) is a complex genetic neurobehavioral/metabolic disorder with an estimated birth incidence of 1:15,000 to 1:25,000 males and females [1]. Clinical features of PWS include hypotonia and poor feeding in infancy; low muscle mass is present throughout life; the accumulation of excess body fat typically begins around age 2 years and continues into adulthood [2]. Ultimately, the central neurological defect of the disease causes PWS patients to sense that they are starving and signals them to significantly increase their caloric intake. This results in hyperphagia, and a progression to morbid obesity if the caloric intake is not carefully managed [2]. Intellectual disability, growth hormone deficiency, behavioral problems, including aggressive and threatening behaviors, and neuroendocrine abnormalities are also characteristic of PWS [1]. The death rate among PWS patients is markedly elevated, with a 3% annual death rate across all ages [3]. According to a 2014 survey of parents and caregivers of PWS patients, reducing hunger and improving food-related behaviors were the most important unmet needs in PWS that could be addressed in the development of a new therapeutic [4]. There are no approved therapeutics for the treatment of hyperphagia in PWS.

### DCCR (diazoxide choline controlled-release tablet)

Diazoxide choline, a new chemical entity, is a benzothiadiazine that acts by stimulating ion flux through ATP-sensitive K+ channels (KATP). It is the choline salt of diazoxide, which is currently used as a treatment of infants, children and adults with hyperinsulinemic hypoglycemia. DCCR is diazoxide choline, formulated as an oral once-a-day extended-release tablet.

### Mode of action of DCCR in PWS

**Fig 1:**
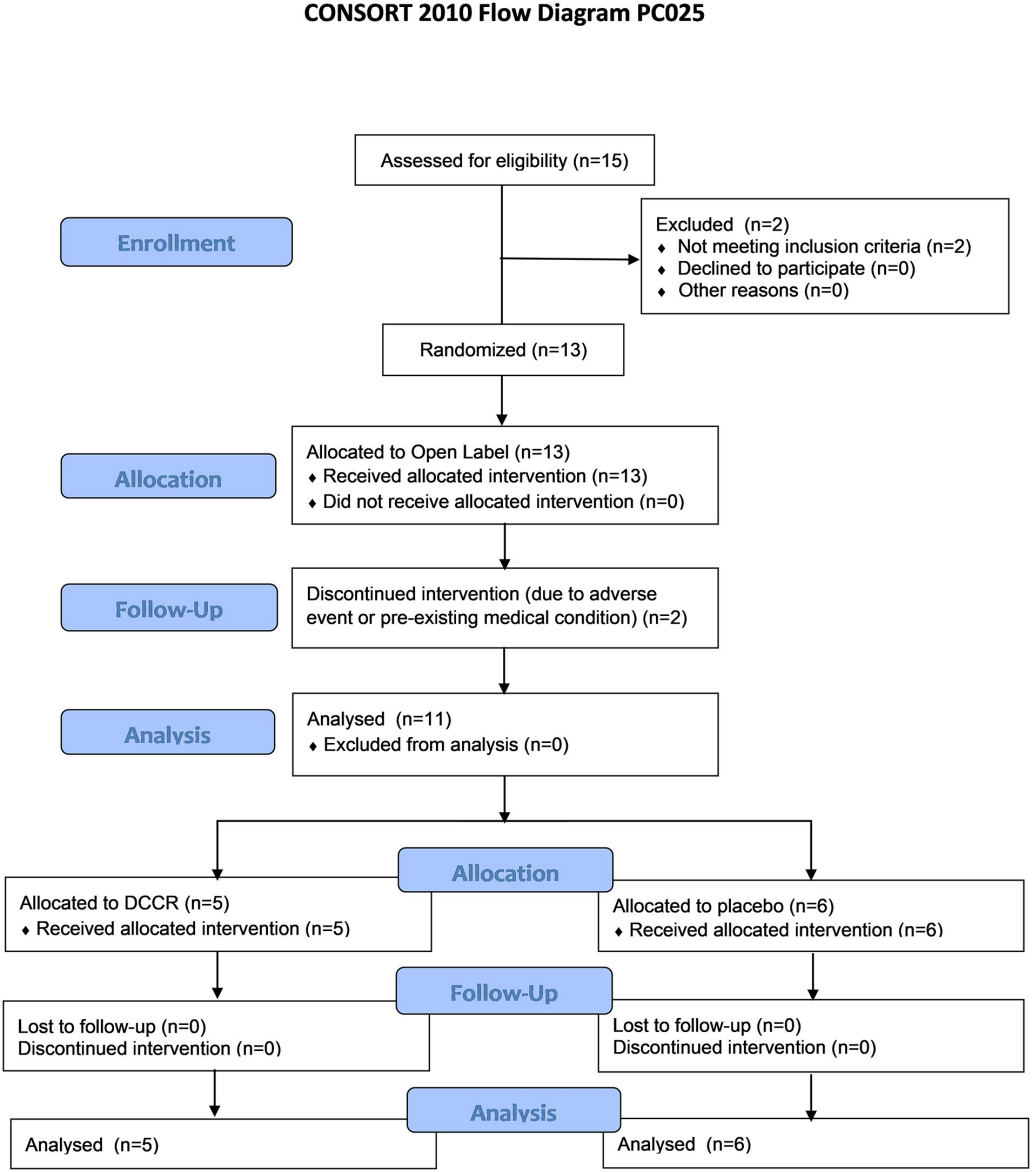
Consort flow diagram for clinical study PC025.

The hyperphagia signal in PWS likely occurs due to the dysregulation of Neuropeptide Y (NPY)/Agouti Related Protein (AgRP)/Gamma-aminobutyric acid (GABA) (NAG) neurons, which appears to be associated with the deletion of SNORD116 in the PWS critical region. This dysregulation results in marked elevations in the synthesis and secretion of NPY, the most potent endogenous neuropeptide by NAG neurons. NAG neurons also synthesize and secrete AgRP and GABA. AgRP colocalizes to the same secretory vesicles in NAG neurons as does NPY, while GABA may be packaged in distinct secretory vesicles. Leptin regulates the NAG neurons through activation of adenosine triphosphate (ATP)-sensitive potassium channels (K_ATP_) [5–8] which serves to hyperpolarize the resting membrane potential, limiting the release of NPY by these neurons. In NAG neurons from adult animals, diazoxide more extensively hyperpolarized the resting energy potential of NAG neurons than does leptin [6]. Diazoxide readily crosses the blood-brain barrier [9], and thus orally-administered diazoxide choline can effectively agonize the K_ATP_ channels in the NAG neurons of the hypothalamus. Agonizing the K_ATP_ channel in these neurons effectively supplements the regulatory effects of leptin, reducing the secretion of NPY and likely AgRP and GABA, thus blunting the hyperphagia signal (Fig 1).

There is strong evidence that activation of NAG neurons results in insulin resistance and impaired glucose tolerance [10]. Inhibiting these neurons by agonizing the K_ATP_ channel, has the potential to improve insulin sensitivity and improve glucose tolerance.

Increases in secretion of NPY by NAG neurons, via hepatic sympathetic innervation, stimulates an increase in very low density lipoprotein (VLDL) -triglyceride secretion by the liver resulting in increased circulating triglycerides and cholesterol [11, 12]. Agonizing the K_ATP_ channel in NAG neurons with a product such as diazoxide choline is expected to result in reduced NPY secretion, reduced hepatic secretion of VLDL and reduced circulating triglycerides and cholesterol.

In parallel, DCCR administration can reduce hyperinsulinemia in treated patients, limiting the deposition of consumed calories as body fat, making them available to meet current energy needs and reducing the contribution of hyperinsulinemia to hyperphagic drive.

## Materials and methods

### Clinical trial design

The clinical study was approved by the University of California Irvine Institutional Review Board. Clinical study PC025 was a single center Phase II study which included a 10-week Open-Label Treatment Period followed by a 4-week Double-Blind, Placebo-Controlled, Randomized Withdrawal Treatment Period (Double-Blind Treatment Period) and was conducted at the University of California, Irvine (Table 1). Patients were initiated on a once-daily oral DCCR dose of approximately 1.5 mg/kg (maximum starting dose of 145 mg) and were to be titrated every 2 weeks to approximately 2.4 mg/kg, 3.3 mg/kg, 4.2 mg/kg, and 5.1 mg/kg (or to a maximum dose of 507.5 mg, whichever was less) at the discretion of the investigator. Any patient who showed any increase in resting energy expenditure and/or any reduction in hyperphagia from Baseline through Week 6 or Week 8 was designated a Responder (pre-defined criteria in the protocol) and eligible to be randomized in the Double-Blind Treatment Period. During the Double-Blind Treatment Period all individuals designated as Responders were to be randomized in a 1:1 ratio either to continue on active treatment at the dose they were treated with at Week 8 or to the placebo equivalent of that dose for an additional 4 weeks. Placebo tablets for each dose strength were identical in weight, shape and appearance to the DCCR active tablets. Patients not designated a Responder (i.e., Non-Responder) were to continue on open-label treatment for an additional 4 weeks. Subjects were randomly assigned to the respective arms by an unblinded pharmacist using a randomly generated sequence of assignment. All other study personnel, the sponsor, the subject and the subject’s caregiver remained blinded to the treatment assignment. Screening started on June 10, 2014 and the last subject last visit took place on April 23, 2015. The trial completed per PC025 protocol. The trial was registered on www.clinicaltrials.gov, identifier NCT02034071.

**Table 1:** Clinical study schematic.

Screening (4 weeks)	Open-Label Treatment Period (10 weeks)						Double-Blind, Placebo-Controlled, Randomized Withdrawal Extension (4 weeks)
Visit 1	Baseline Week 0 Visit 2	Week 2 Visit 3	Week 4 Visit 4	Week 6 Visit 5	Week 8 Visit 6	Week 10 Visit 7	Week 14 Visit 8
	1.5 mg/kg	Up to 2.4 mg/kg	Up to 3.3 mg/kg	Up to 4.2 mg/kg	Up to 5.1 mg/kg	Week 8 dose or Placebo equivalent for Responders Week 8 dose for Non-Responders	

The primary objective of the study was to evaluate the safety of multiple doses of DCCR in PWS patients. The secondary objectives were to evaluate the effect of DCCR treatment on hyperphagia and resting energy expenditure in PWS patients.

#### Key inclusion criteria

- Ability to follow verbal and written instructions with or without assistance from caregiver
- Informed consent form signed by the subject or their legal guardian
- Male and female patients 10 to 22 years of age, inclusive at the time of dosing
- Genetically-confirmed Prader-Willi syndrome
- Body Mass Index (BMI) exceeds the 95^th^ percentile of the age specific Body Mass Index (BMI) value on the CDC BMI charts or percent body fat ≥ 35%

#### Key exclusion criteria

- Administration of investigational drugs within 1 month prior to Screening Visit
- Allergic to or significant intolerance of diazoxide, thiazides or sulfonamides.
- Anticipated transition in their care from family home to group home or other similar potentially disruptive changes
- Known type 1 diabetes mellitus
- Supine systolic blood pressure > 160 mm HG and/or supine diastolic blood pressure > 100 mm Hg at the Screen Visit

#### Methods

The schedule of assessments is shown in Table 2. Hyperphagia was measured in the study using a 9-question Modified Dykens questionnaire [13], posed to the parent or caregiver which utilized a 2-week recall period. Each answer was associated with a numeric value which were summed across questions to yield a total hyperphagia score (range 0–34). Data from hyperphagia assessments at Baseline (defined as the average of hyperphagia scores at the Screening and Baseline visits) and the end of the Open-Label Treatment Period and the end of the Double-Blind Treatment Period were used in the analysis. Changes in body fat and lean body mass were measured using dual-energy x-ray absorptiometry (DEXA) at Baseline and at the end of the Open-Label Treatment Period, but not the end of the Double-Blind Treatment Period. Behavioral assessments were conducted using a questionnaire from the Prader-Willi Syndrome Natural History Study [14]. The questionnaire assessed the presence or absence of 23 PWS-associated behaviors (grouped into 4 categories) at Baseline and at the end of the Open-Label Treatment Period, but not the end of the Double-Blind Treatment Period. Resting energy expenditure (REE) and respiratory quotient (RQ) were measured by indirect calorimetry. Other assessments were completed by standard means [15].

**Table 2:** Schedule of assessments.

Period (duration)	Open Label Treatment Period (10 Weeks)						Double-Blind Period (4 Weeks)
Week	0 (Baseline)	2	4	6	8	10	14
DEXA	X					X	
Behavioral questionnaire	X					X	
Fasting glucose	X	X	X	X	X	X	X
Ghrelin, leptin, triglycerides, Total C, LDL-C, HDL-C, non-HDL-C, comprehensive metabolic panel	X					X	X
Hyperphagia questionnaire	X	X	X	X	X	X	X
Resting energy expenditure, respiratory quotient	X		X		X	X	X
Pharmacokinetic blood sampling						X	X

#### Statistical analysis

All endpoints measured during the Open-Label Treatment Period, except for changes in behavior were analyzed by paired t-tests, as was the change within arms from Baseline through the end of the Double-Blind Treatment Period. Analysis of covariance (ANCOVA) was performed on the endpoints measured during the Double-Blind, Treatment Period. These models included treatment arm as a factor and the Baseline value of the corresponding endpoint as a covariate. Behavioral changes were analyzed using Pearson’s Chi-Squared Test for goodness of fit using Baseline frequencies as the expected values. Adverse events that were newly arisen in the double-blind extension period were summarized by treatment arm and analyzed by Fisher’s exact test. All of these analyses were preplanned. Hyperphagia change from Baseline to the end of the Double-Blind Treatment Period was also analyzed post-hoc by two-group t-test.

No prior data existed for DCCR in patients with Prader-Willi syndrome, thus the sample size was calculated based on previously published studies in obese subjects on reduced calorie diet where weight loss was observed and attributable almost exclusively to the loss of body fat.

## Results

### Subjects enrolled in the study

Fifteen subjects were screened and 13 subjects were enrolled in the study. All but one of the subjects in the study were cared for at home. One subject resided in a group home during the week and at home on weekends. Subjects were instructed not to change their diet or exercise habits over the course of the study. Growth hormone treated subjects had all been treated with growth hormone for at least 1 year prior to the start of the study and some for more than a decade. Subjects could not initiate or discontinue growth hormone treatment during the study. Ten of the 13 subjects were obese, based on BMI, at baseline, and 3, who met the body fat content criteria for inclusion, were overweight. Table 3 summarizes the baseline characteristics of subjects enrolled in the study and of those subjects who completed the Open-Label Treatment Period and were randomized into the Double-Blind Treatment Period. Per protocol, subjects ages 10–22 could be randomized into the study, however the age of enrolled subjects was 11–21 years.

**Table 3:** Baseline characteristics of enrolled and randomized subjects.

	Enrolled (n = 13)		Completed Open-Label Treatment Period and Randomized into Double-Blind Treatment Period (n = 11)	
Parameter	Statistic	Range	Statistic	Range
**Gender (Male [M]/Female [F])**	8M/5F		6M/5F	
**Age (years)**	15.5±3.0	11–21	15.6±2.9	11–21
**Weight (kg)**	89.6±24.9	56.4–134.1	90.4±25.4	56.4–134.1
**Height (cm)**	153.7±10.9	140–177.4	154.0±11.7	140–177.4
**Percent body fat (%)**	51.7±6.9	36.4–60.7	51.8±6.6	36.4–60.7
**BMI (kg/m**^**2**^**)**	38.1±10.9	24.7–53.5	38.2±10.7	24.7–53.5
**Hyperphagia score**	16.2±8.1	3–29	15.0±8.1	3–29
**Fasting Glucose (mmol/L)**	4.64±0.34	4.05–5.05	4.67±0.33	4.05–5.05
**Fasting Insulin (pmol/L)**	95.4±74.2	13.9–245.9	85.3±62.0	13.9–193.8
**Waist Circumference (cm)**	113.3±20.1	84.2–146.4	113.2±20.4	84.2–146.4
**Ghrelin (ng/L)**	1119.3±482.2	568–2138	1044.9±392.7	568–1731
**Current growth hormone use (Yes [Y]/No [N])**	7Y/6N		7Y/4N	
**PWS subtype (Deletion/ Uniparental disomy [UPD])**	12 Deletion / 1 UPD		10 Deletion / 1 UPD	

### Subject disposition

**Fig 2:**
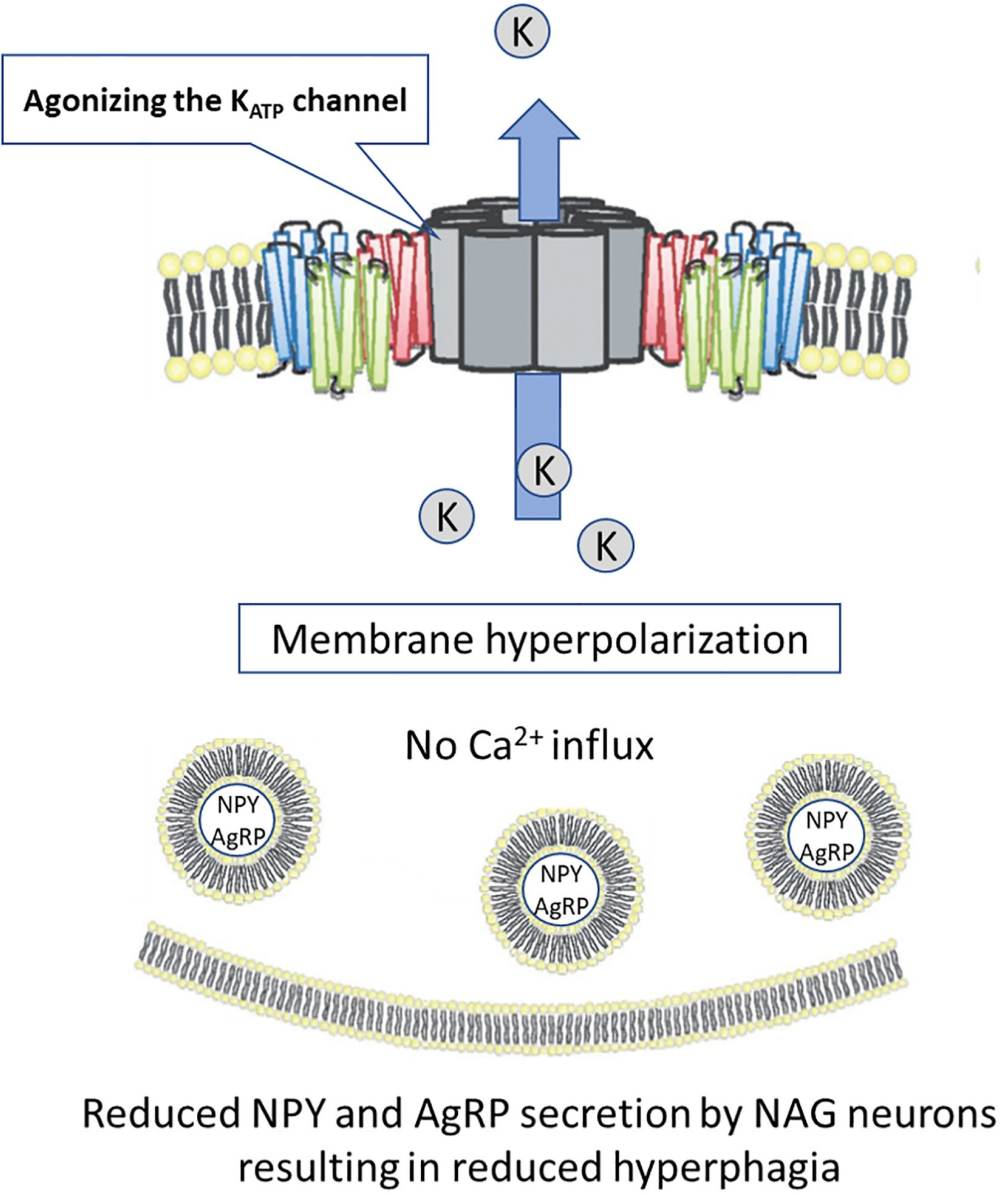
Mode of action of DCCR in hyperphagia.

Of the 15 subjects screened, 13 (86.7%) subjects were enrolled into the study (Fig 2). The Open-Label Treatment Period was completed by 11 of the 13 enrolled subjects (84.6%), and 2 subjects discontinued due to adverse events—one related to DCCR (hyperglycemia) and one unrelated (institutionalization due to a pre-existing psychiatric condition). All 11 subjects who completed the Open-Label Treatment Period were designated Responders, all having shown improvements in hyperphagia and most having shown improvements in REE, and randomized into the Double-Blind Treatment Period. All 11 (100%) subjects who were randomized into the Double-Blind Treatment Period completed the period.

### Dosing

The mg/kg dose was achieved as closely as possible using the available dose strengths of DCCR (72.5 and 145 mg), thus the mg/kg doses are approximate. Of those who completed the study, one subject started and finished the Open-Label Treatment Period on a dose of 1.5 mg/kg, two were titrated to 2.4 mg/kg, four were titrated to 3.3 mg/kg and continued at that dose for the remainder of the study, and the remaining four subjects completed the Open Label Treatment Period at a dose of 4.2 mg/kg. The investigator chose not to titrate subjects to 5.1 mg/kg. A single subject was dosed with 5.1 mg/kg in error for a few days before being dose reduced to 4.2 mg/kg. One of the subjects who completed the Open Label Treatment Period at a dose of 2.4 mg/kg had been treated with a higher dose, but was dose reduced to help manage peripheral edema. Subjects randomized later in the study tended to be titrated to the 4.2 mg/kg dose, as the safety profile of DCCR in PWS patients became better understood.

### Pharmacokinetics

**Fig 3:**
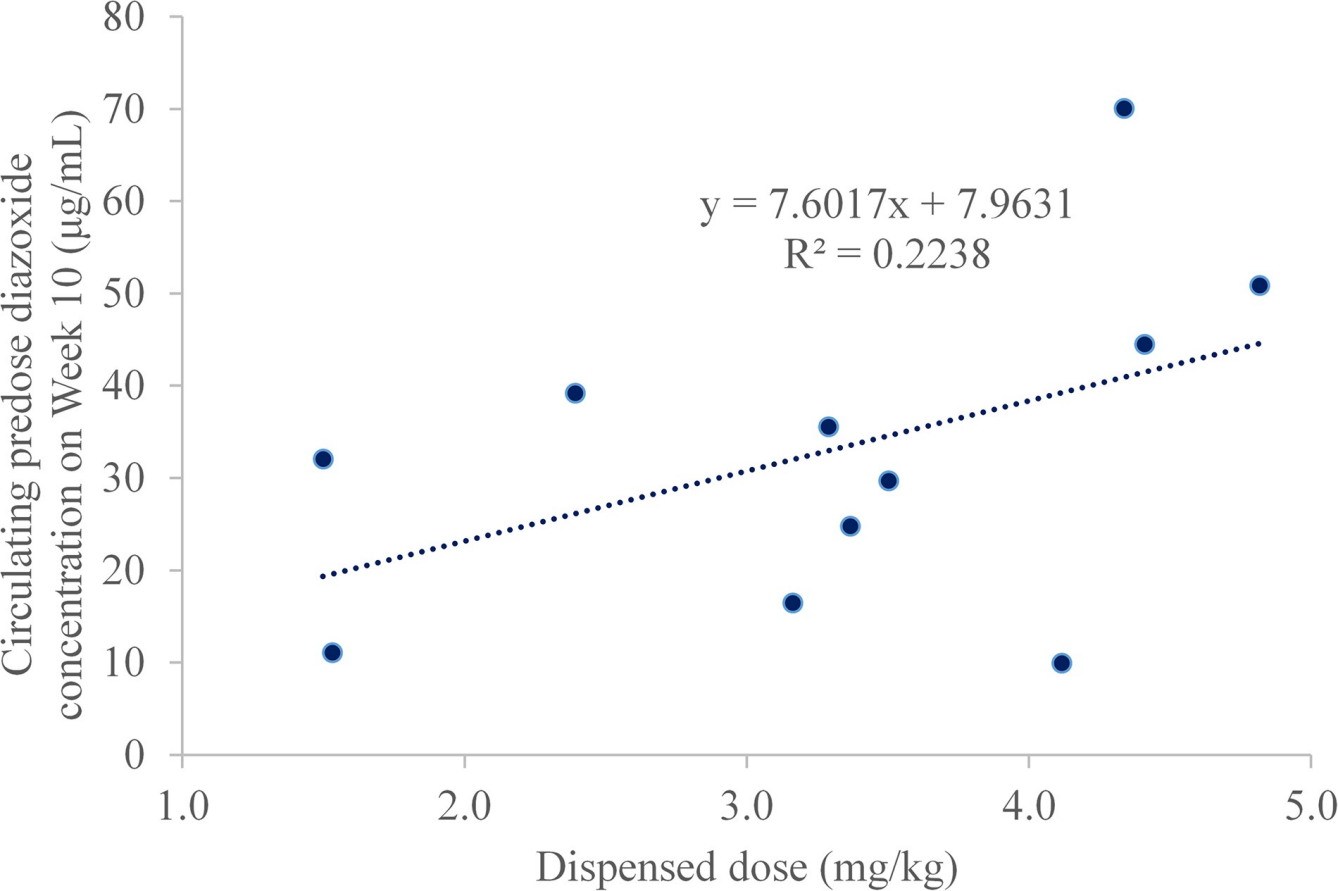
Relationship between predose circulating drug level and dispensed dose week 10.

Pharmacokinetic samples were available for all subjects who completed the Open-Label Period for both the end of the Open Label Treatment Period (Week 10) and the end of the Double-Blind Treatment Period (Week 14). At Week 10, all subjects had circulating drug levels above the limits of quantitation. The mean circulating drug level on Week 10 was 33.13 μg/mL and ranged from 9.94 to 70.1 μg/mL. No statistical analysis of pharmacokinetic data was completed. At Week 10, there was a linear relationship between circulating drug level and the actual mg/kg dose (Fig 3). These results were similar to those observed in obese subjects treated with DCCR in a previously completed study. At Week 14, all of the placebo treated subjects had circulating drug levels below the limits of detection, as did one subject in the DCCR group who, due to an extended family vacation overseas, was not able to return for the final study visit until 2 weeks after their last dose of study drug.

### Changes in hyperphagia

**Fig 4:**
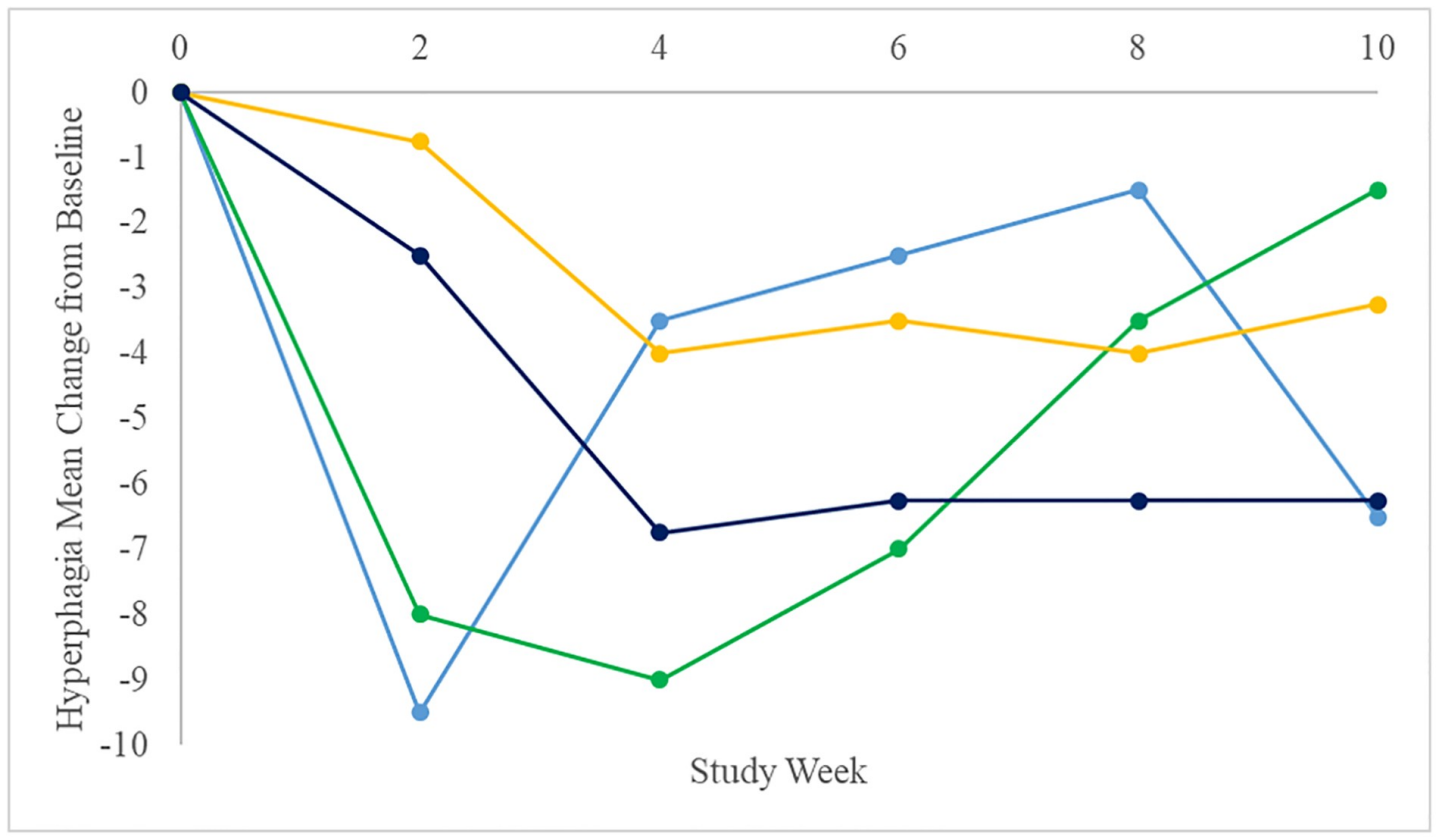
Hyperphagia mean change from baseline during the Open-Label Treatment Period by final dose. 1.5 mg/kg (n = 1) shown in light blue, 2.4 mg/kg (n = 2) shown in green, 3.3 mg/kg (n = 4) shown in yellow, and ≥ 4.2 mg/kg (n = 4) shown in dark blue.

The mean baseline hyperphagia score for all subjects enrolled in the study was 16.2 and since there was no hyperphagia criteria for inclusion, ranged from 3 to 29. After 2 weeks of treatment, there was a significant reduction in hyperphagia score (-3.31, n = 13, p = 0.02); the statistically significant reduction in hyperphagia score was sustained through the remainder of the Open-Label Treatment Period. There was a statistically significant improvement in hyperphagia at the end of the Open-Label Treatment Period (-4.32, n = 11, p = 0.006). Subjects reaching a final dose of either 1.5 mg/kg or 2.4 mg/kg, while displaying a marked initial response to treatment, were unable to maintain the improvements observed at 2 or 4 weeks of treatment through the end of the Open-Label Treatment Period (Fig 4). In contrast, subjects reaching a final dose of either 3.3 mg/kg or 4.2 mg/kg were able to effectively maintain the improvement in hyperphagia achieved upon reaching their final dose (at 4 and 6 weeks, respectively) through the end of the Open-Label Treatment Period on Week 10 (Fig 4).

**Fig 5:**
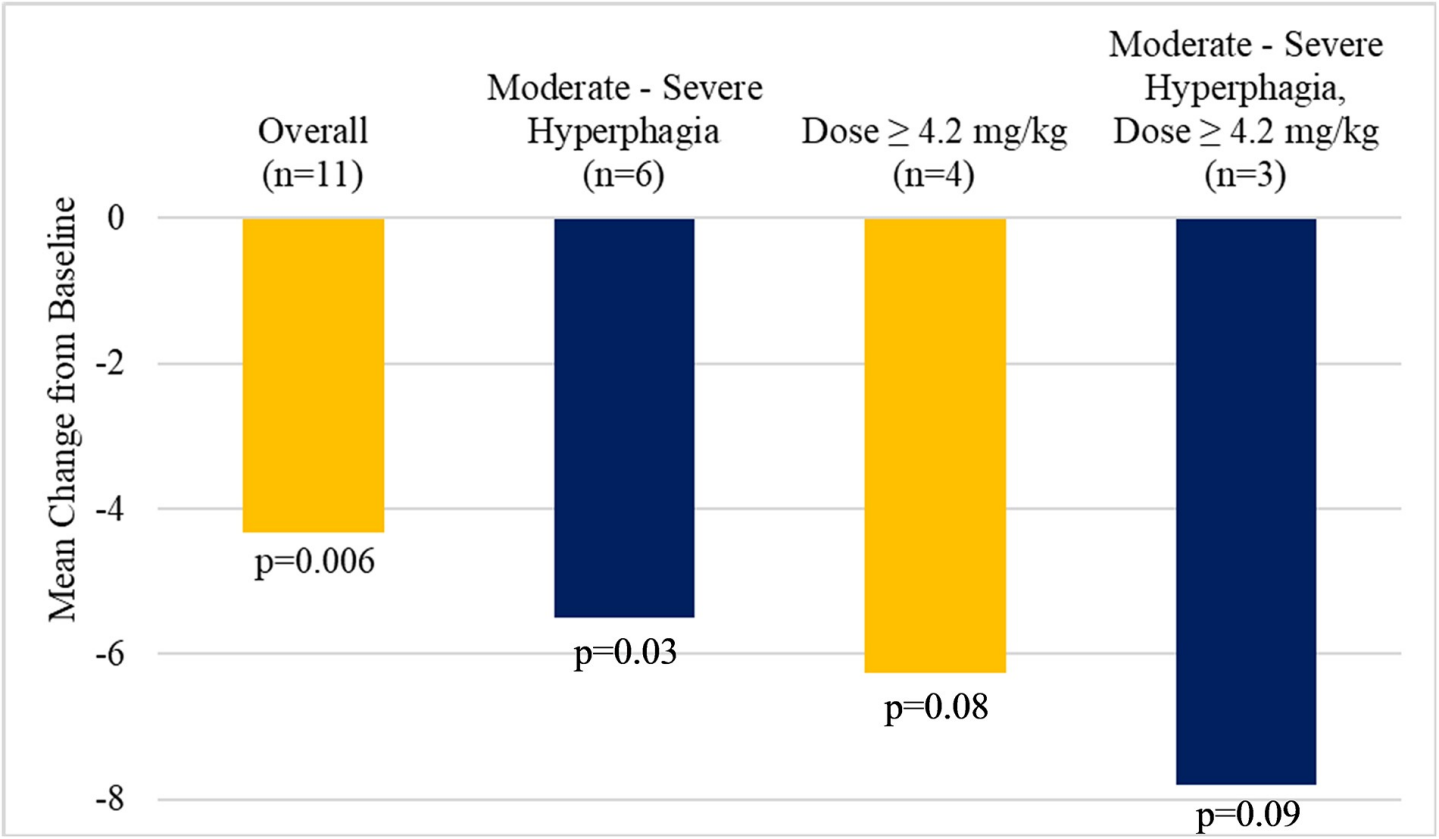
Hyperphagia change from baseline to the end of Open-Label Treatment Overall, High Dose and/or moderate to severe hyperphagia.

**Fig 6:**
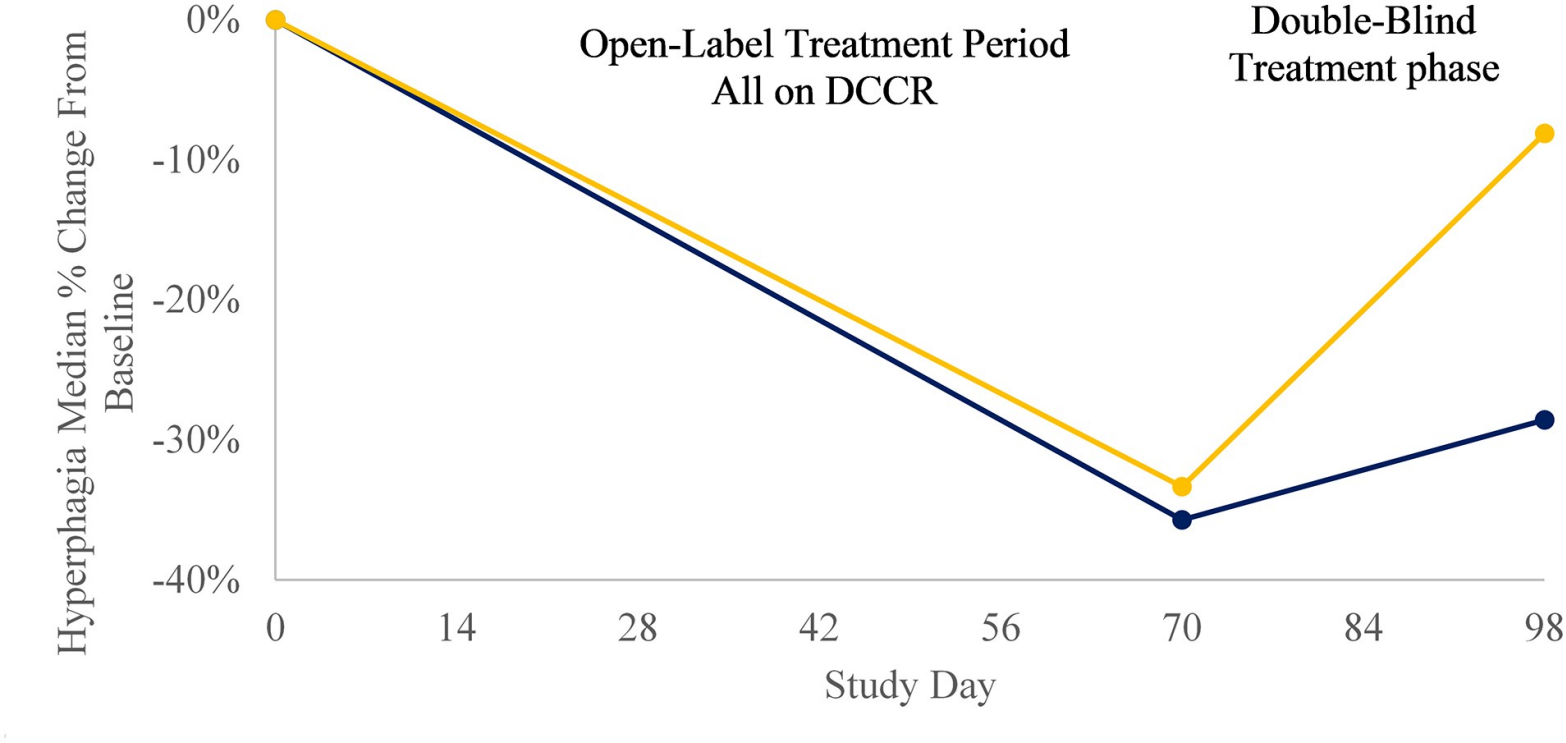
Changes in hyperphagia in subjects with moderate to severe baseline hyperphagia. DCCR (n = 3) shown in dark blue, Placebo (n = 3) shown in yellow.

Growth-hormone treated subjects appeared to show greater improvements in hyperphagia (-5.07, n = 7, p = 0.03) than did growth-hormone naïve subjects (-3.0, n = 4, p = 0.09) predominantly due to their lower mean Baseline hyperphagia (15.9 versus 13.3, respectively). The greatest improvement in hyperphagia were observed in subjects who had moderate to severe hyperphagia at Baseline and were treated with the highest dose (Fig 5). All late-stage trials in Prader-Willi syndrome where hyperphagia is the primary endpoint are focused on subjects with moderate to severe hyperphagia. Fig 6 shows the median response to treatment in the subset of subjects in this study with moderate to severe hyperphagia. In both those subjects randomized to DCCR in the Double-Blind Treatment Period and those subjects randomized to placebo in the Double-Blind Treatment Period showed a marked median improvement in hyperphagia during the Open-Label Treatment Period. During the Double-Blind Treatment Period Placebo-treated subjects regressed back towards Baseline while those subjects continuing on DCCR during the Double-Blind Treatment Period continued to show marked median improvements in hyperphagia.

With 5 DCCR-treated and 6 placebo treated subjects, the difference in median hyperphagia score between the arms from Baseline through the end of the Double-Blind Treatment Period was not significant when the data was analyzed by ANCOVA (p = 0.1076), while the two-group T-test comparing hyperphagia change from Baseline in the arms was statistically significant (p = 0.05). The mean improvement from Baseline in hyperphagia in those who were randomized to DCCR in the Double-Blind Treatment Period persisted through the end of the study (-4.8, n = 5, p = 0.012). There was a tendency for placebo treated subjects to regress back towards there Baseline hyperphagia scores, with the mean change from Baseline to the end of the Double-Blind Treatment Period for placebo treated subjects being -1.92 (n = 6, p = 0.105).

In addition to the hyperphagia scores, verbatim comments by parents were supportive of a marked impact on hyperphagia. Parents noted their PWS children skipping meals, declining food offered to them even after an extended overnight fast or had stopped asking about meals.

### Changes in body composition

**Fig 7:**
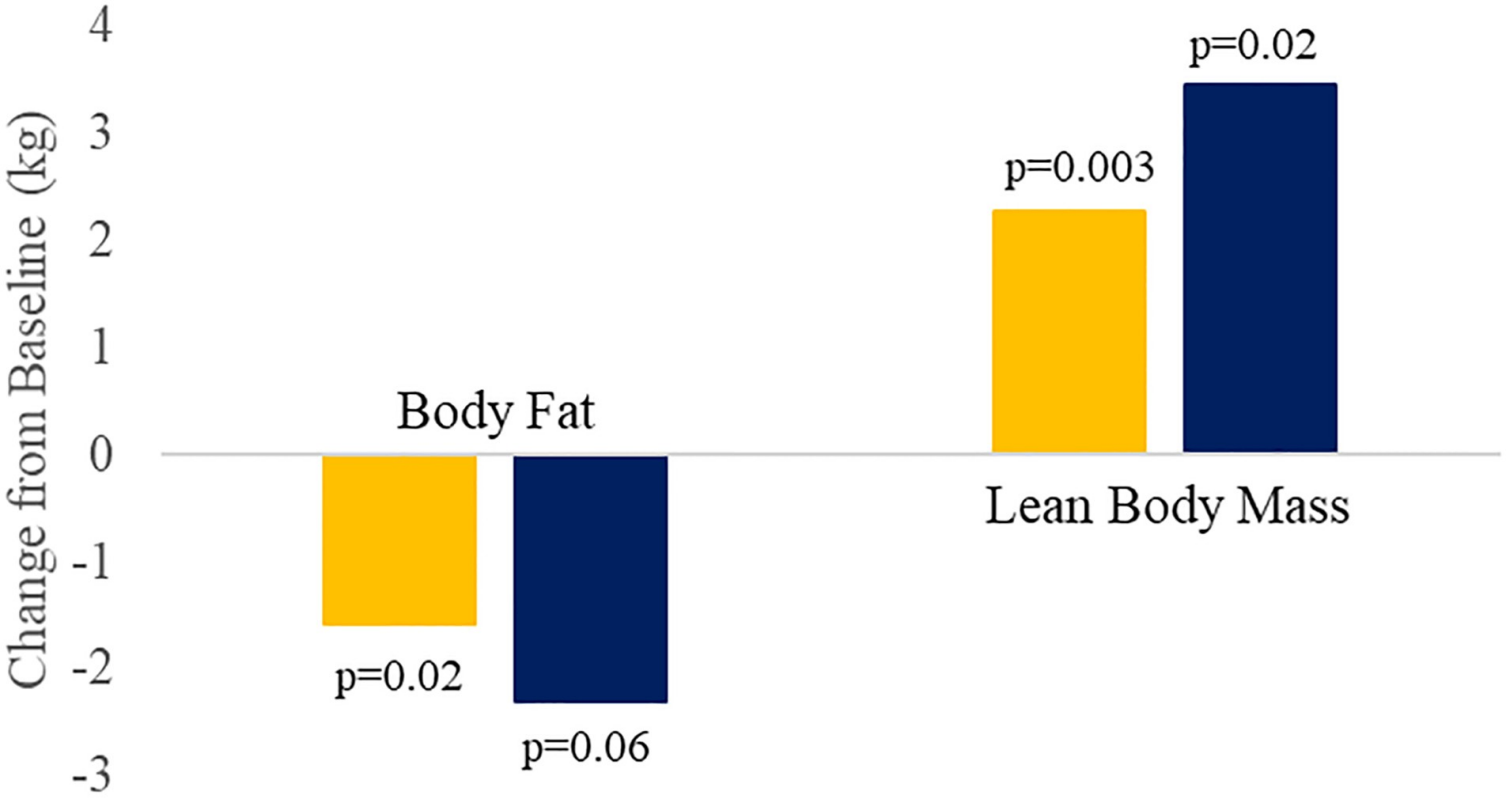
Body fat and lean body mass change from baseline to the end of the Open-Label Treatment Overall and High Dose. Overall (n = 11) shown in yellow and ≥ 4.2 mg/kg (n = 4) shown in dark blue.

Treatment with DCCR for 10 weeks had a significant impact on body composition including reductions in body fat, increases in lean body mass and a marked increase in lean body mass to body fat ratio. About 75% of the subjects experienced reductions in fat mass, more than 90% experienced increases in lean body mass, while 100% experienced increases in lean body mass/fat mass ratio. All of the changes in body composition showed strong dose dependence. Table 4 and Fig 7 summarize the changes in body composition.

**Table 4:** Changes from baseline in body composition by DEXA during the Open-Label Treatment Period.

Parameter	n	Change	p-value*
Body fat percent	11	- 2.2%	0.01
Body fat mass	11	-1.59 kg	0.02
Lean body mass	11	2.26 kg	0.003
Lean body mass/fat mass ratio	11	9.63%	0.002
Weight	11	0.47 kg	0.32

*Paired t-test

Because the PWS subjects enrolled in this study had almost equal lean body mass and fat mass at Baseline, parallel increases in lean body mass, fluid retention and reductions in body fat resulted in almost no net change in weight. A DEXA was not conducted at the end of the Double-Blind Treatment Period.

### Changes in weight and waist circumference

There was no significant change in weight from Baseline through the end of either the Open-Label Treatment Period, or the end of Double-Blind Treatment Period, likely due to the significant decrease in fat mass and a corresponding increase in lean body mass. Waist circumference was significantly reduced during the Open-Label Treatment Period (-3.45 cm, p = 0.006), suggesting there was loss of visceral fat. During the Double-Blind Treatment Period, waist circumference increased incrementally in the placebo arm (0.25 cm) while there was a marked reduction in those randomized to DCCR (-2.93 cm, p = 0.11 for the comparison to placebo).

### Behavioral changes

**Fig 8:**
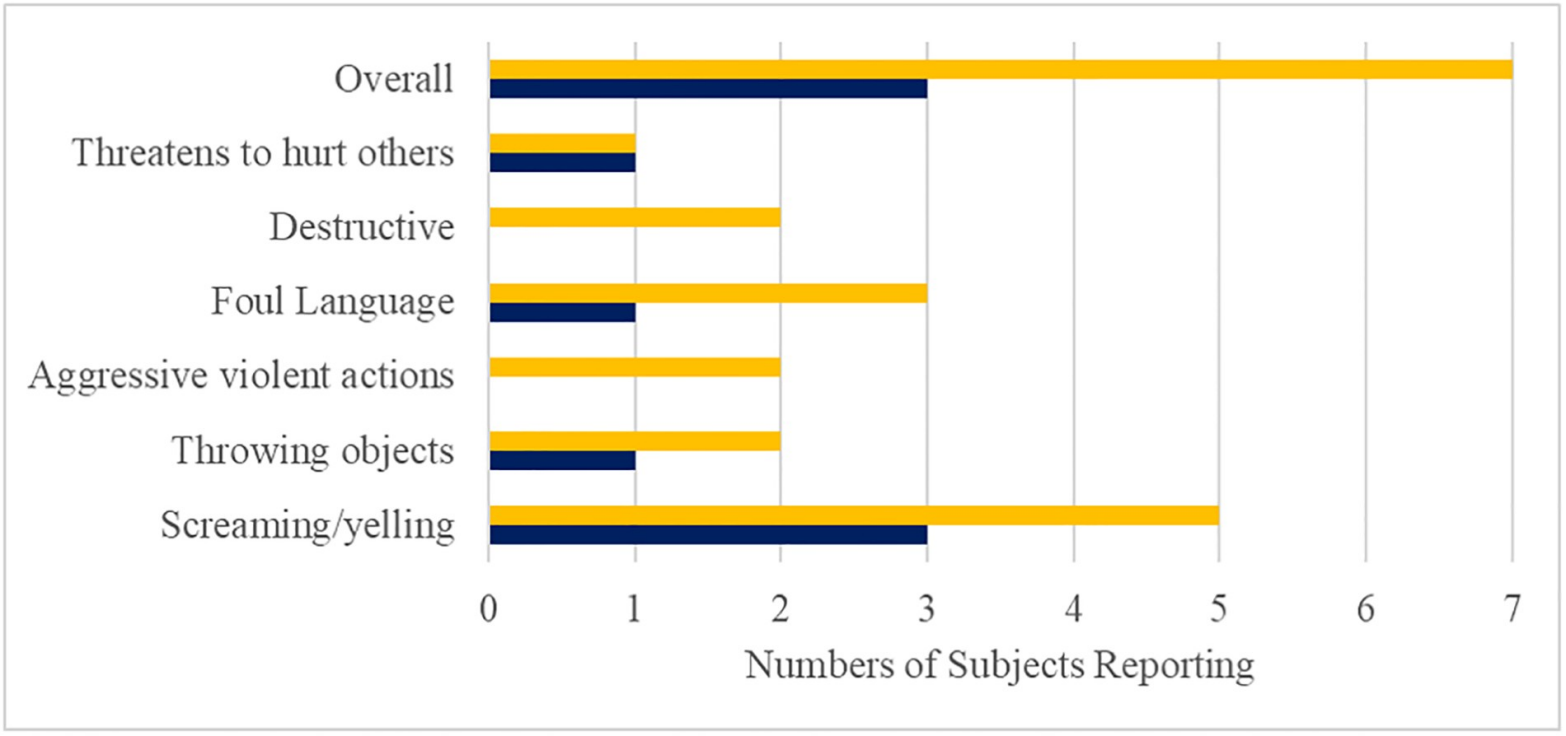
Change numbers of subjects reporting aggressive, threatening and destructive behaviors from baseline to end of the Open-Label Treatment. Baseline shown in yellow and End of Open-Label shown in dark blue.

The behaviors assessed at Baseline and at the end of the Open-Label Treatment Period using the behavioral questionnaire grouped into 4 categories: (1) aggressive, threatening and destructive behaviors; (2) self-injurious behaviors; (3) compulsive behaviors; and (4) other behaviors. There was a marked improvement in aggressive, threatening and destructive behaviors (70% of subjects displayed one or more of these behaviors at Baseline vs. 30% at the end of the Open-Label Treatment Period (chi-square test p = 0.01, Fig 8). Most behaviors in the category were reduced associated with DCCR treatment, and some stopped destructive behaviors and aggressive, violent actions completely. These behaviors contribute substantially to diminished quality of life of PWS families and are the primary reason PWS patients are moved from home care into institutional care. There was a reduction in self-injurious behavior (numbers of reported behaviors at Baseline and the end of the Open-Label Treatment Period 13, and 9 respectively; p = 0.23), there were no improvements in the compulsive behaviors or other behaviors categories. Behavioral changes were not assessed at the end of the Double-Blind Treatment Period.

### Changes in lipids

The PWS subjects enrolled in this study did not present with marked dyslipidemia at Baseline. Changes in fasting lipids are shown in Table 5. Nonetheless, consistent with previous studies with DCCR, 10 weeks of treatment resulted in marked changes in lipids including reductions in triglycerides, total cholesterol, LDL cholesterol and non-HDL cholesterol.

**Table 5:** Changes in fasting lipids.

Parameter	Open-Label Treatment Period			Double-Blind Treatment Period				
				DCCR		Placebo		p-value*
	n	Change from Baseline	p-value	n	Change from Baseline	n	Change from Baseline	
Triglycerides	7	-24.2%	0.15	4	-40.3%	6	-31.5%	0.046
Total Cholesterol	8	-6.3%	0.07	3	-8.8%	6	2.7%	0.10
Non-HDL Cholesterol	8	-11.0%	0.03	4	-4.3%	6	5.3%	0.52
LDL Cholesterol	8	-12.5%	0.03	4	-7.2%	6	-8.1%	0.38

*Based on ANCOVA

During the Double-Blind Treatment Period there were small further improvements in the DCCR arm for triglycerides and total Cholesterol, but not for the other lipid parameters, and a regression towards Baseline in the placebo arm in total cholesterol and non-HDL cholesterol.).

### Changes in leptin and ghrelin

At the end of the Open-Label Treatment Period, there was a 22% reduction in leptin (-12.72 ng/mL, p = 0.007), while ghrelin was virtually unchanged over 10 weeks of treatment with DCCR (9.2 pg/mL, p = 0.93). Thus, it appears that a significant reduction in hyperphagia in PWS patients can be realized in the absence of a change in ghrelin. The change from Baseline to the end of the Double-Blind Treatment Period was not significant in either arm for either leptin or ghrelin.

### Changes in resting energy expenditure and respiratory quotient

The measurements for resting energy expenditure and respiratory quotient were quite variable from visit to visit, suggesting there may be limitations to the use of this method in patients with PWS. The variability in the measurements may have accounted for the apparent improvements in REE at some visits for some patients. Resting energy expenditure (REE) and respiratory quotient (RQ) appeared to be unchanged from Baseline through the end of the Open-Label Treatment Period (REE 0.3, n = 11, p = 0.947; RQ 0.001, n = 11, p = 0.97). Similarly, there were very small, non-significant changes in REE (DCCR: -10.6, n = 5; Placebo: -4.7, n = 6; p = 0.5) and RQ (DCCRL 0.03, n = 5; Placebo: -0.005, n = 6); p = 0.90) through the end of the Double-Blind Treatment Period.

### Adverse events

Nearly all of the adverse events were of mild to moderate severity and resolved while dosing continued. The frequency of treatment emergent adverse events in the Double-Blind Treatment Period was similar between the arms. Fisher’s exact test comparing numbers of subjects experiencing a treatment emergent adverse event confirmed that there was no significant difference between the arms (p > 0.9). Many of the adverse events were common medical complications of PWS including: respiratory infections, constipation, peripheral edema and hypersomnia. Drug-related, Treatment-Emergent Adverse Events (TEAEs) that occurred during the Open-Label Treatment Period are summarized in Table 6 and that occurred during the Double-Blind Treatment Period in Table 7.

**Table 6:** Drug-related, Treatment-Emergent Adverse Events (TEAEs) that occurred during the Open-Label Treatment Period.

System Organ Class Preferred Term	Drug-Related TEAEs (N = 13)	Severe Drug-Related TEAEs (N = 13)
Total Number of Subjects with TEAEs	9 (69.2%)	3 (23.1%)
*Metabolism and nutrition disorders*	8 (61.5%)	1 (7.7%)
Hyperglycemia	4 (30.8%)	1 (7.7%)
Glucose tolerance impaired	4 (30.8%)	--
Polydipsia	1 (7.7%)	--
Type 2 diabetes mellitus	1 (7.7%)	--
*General disorders and administration site* conditions	6 (46.2%)	2 (15.4%)
Peripheral edema	5 (38.5%)	2 (15.4%)
Face edema	1 (7.7%)	--
Pain	1 (7.7%)	--
Thirst	1 (7.7%)	--
*Nervous system disorders*	1 (7.7%)	--
Headache	1 (7.7%)	--
*Gastrointestinal disorders*	1 (7.7%)	--
Constipation	1 (7.7%)	--
*Respiratory*, *thoracic and mediastinal disorders*	1 (7.7%)	--
Dyspnea	1 (7.7%)	--
*Skin and subcutaneous tissue disorders*	1 (7.7%)	--
Hirsutism	1 (7.7%)	--
Hyperhidrosis	1 (7.7%)	--
*Renal and urinary disorders*	1 (7.7%)	--
Polyuria	1 (7.7%)	--

**Table 7:** Drug-related TEAEs that occurred during the Double-Blind Treatment Period.

System Organ Class Preferred Term	Placebo (N = 6)	DCCR (N = 5)
Total Number of Subjects with TEAEs	3 (50%)	1 (20%)
*Gastrointestinal disorders*	--	1 (20%)
Diarrhea	--	1 (20%)
*General disorders and administration site* conditions	1 (16.7%)	--
Peripheral edema	1 (16.7%)	--
*Investigations*	--	1 (20%)
Blood cholesterol increased	--	1 (20%)
*Nervous system disorders*	1 (16.7%)	--
Headache	1 (16.7%)	--
*Psychiatric disorders*	1 (16.7%)	--
Dermatillomania	1 (16.7%)	--
*Respiratory*, *thoracic and mediastinal disorders*	--	1 (20%)
Rhinorrhoea	--	1 (20%)
*Skin and subcutaneous tissue disorders*	1 (16.7%)	--
Hair colour changes	1 (16.7%)	--

Peripheral edema was a common adverse event of DCCR in prior studies, and it was in this study as well, occurring in two subjects who presented with it at Baseline or Screening and in three other subjects on treatment. In two subjects with severe peripheral edema (both of whom presented with it at baseline), one was prescribed a diuretic for 5 days and had a dose reduction; the second subject had a DCCR dose reduction. In the other 3 subjects, all with mild peripheral edema, the edema resolved without the need for drug holiday, dose adjustment or use of diuretic. No subject who experienced peripheral edema discontinued from treatment or the study.

**Fig 9:**
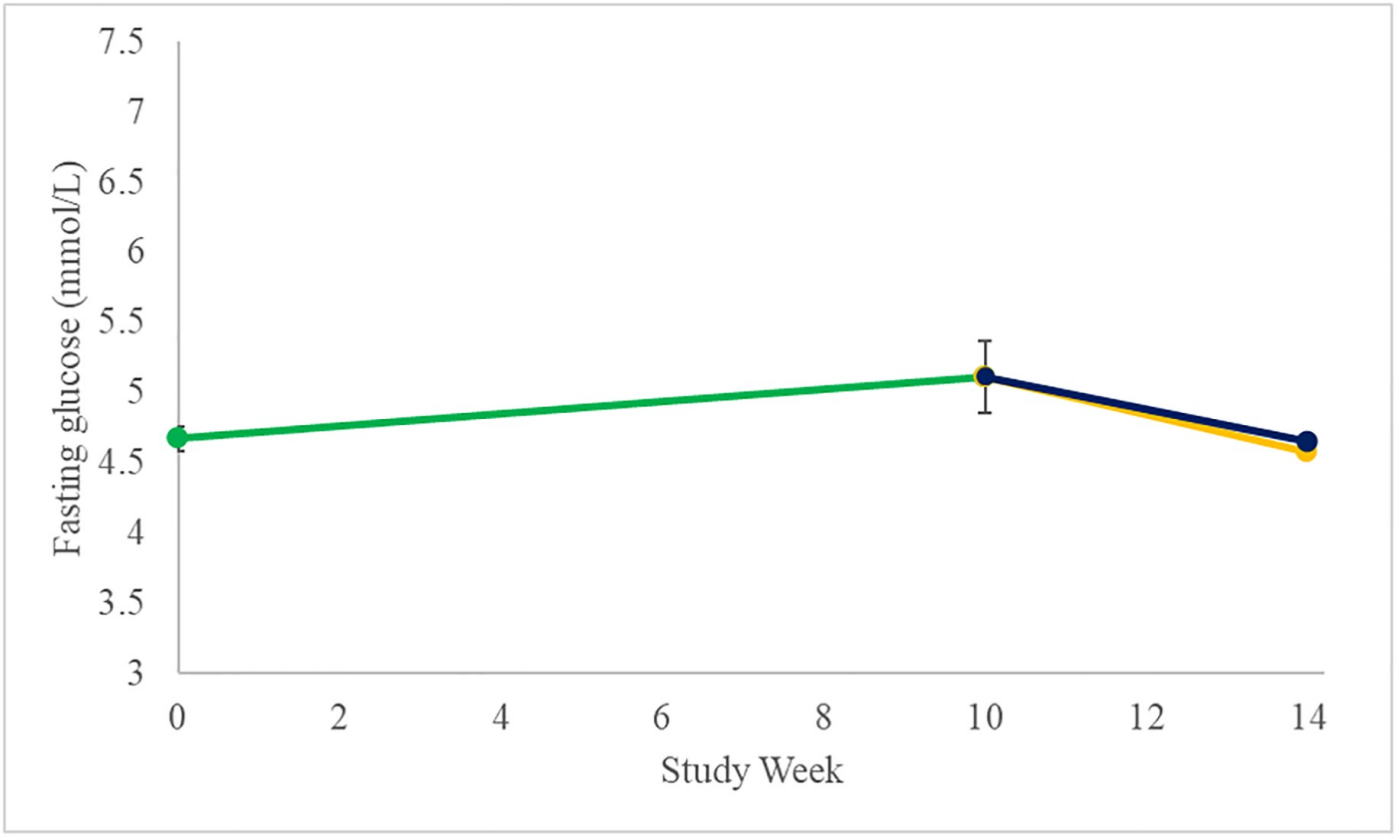
Changes in fasting glucose (Mean±SEM). Open-Label shown in green, Placebo shown in yellow, and DCCR shown in dark blue.

In previously completed studies with DCCR, fasting glucose tended to rise during titration of subjects to target dose and then regress towards baseline as subjects continued on treatment. This pattern was evident in this study as well (Fig 9). On average there was a 9.4% increase in fasting plasma glucose (FPG) during the 10 weeks of Open-Label Treatment (n = 11, p = 0.10). At the end of the Double-Blind Treatment Period, the change from Baseline for fasting glucose in the DCCR arm was 0.02 mmol/L (0.4%, p = 0.86) and was -0.13 mmol/L (-2.8%, p = 0.40) in the placebo arm; the difference between groups was not statistically significant (p = 0.48). All subjects who continued on treatment with DCCR through the Double-Blind Treatment Period were normoglycemic at the end of the study.

**Fig 10:**
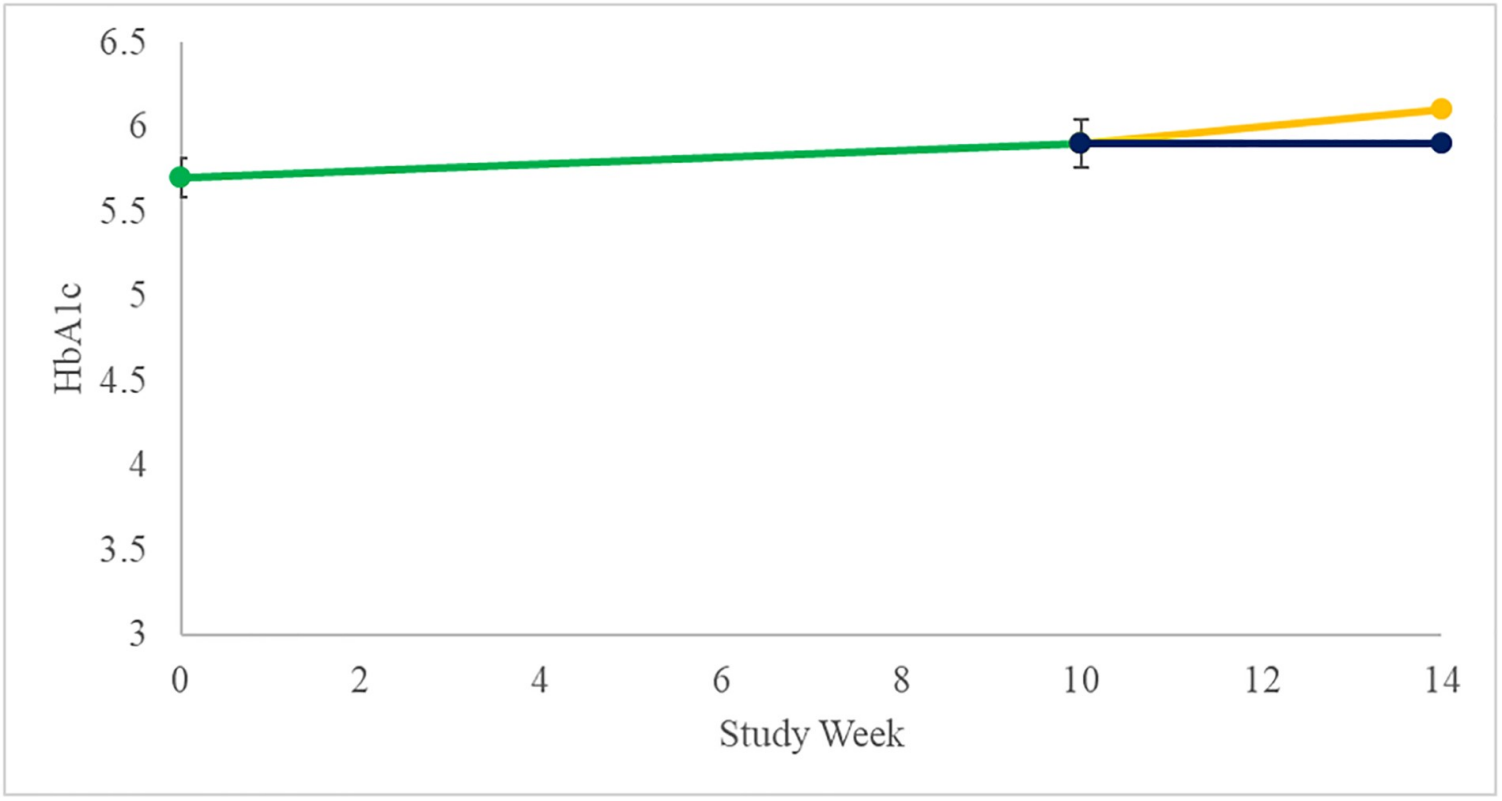
Change in HbA1c (Mean±SEM). Open-Label shown in green, Placebo shown in yellow, and DCCR shown in dark blue.

Associated with the rise in fasting glucose, there was an increase in mean HbA1c from Baseline to the end of the Open-Label Treatment Period, however this was not significant (0.2%, p = 0.06). There was no difference between the treatment arms in mean change from Baseline to the end of the Double-Blind Treatment Period (p = 0.99); however, the mean increase from Baseline in HbA1c in the placebo treated subjects did reach statistical significance (0.25%; p = 0.04, n = 6) (Fig 10).

During the Open-Label Treatment Period HOMA-IR, a measure of insulin resistance, dropped 40.2%, from 2.61 to 1.56 (n = 10) but this decrease was not statistically significant (p = 0.19).

Two subjects withdrew from the study prior to completion of the Open-Label Treatment Period. The first had a history of repeated psychiatric episodes, which continued in the study, and one of which resulted in an overnight hospitalization. Due to challenges managing the subject in the home, he was withdrawn from the study and placed in a PWS group home. The investigator assessed this to be unrelated to study drug.

The second subject had a baseline HbA1c value of 6.2, suggestive of impaired glucose tolerance as well as a family history of Type 2 Diabetes Mellitus. He showed a progressive compromising of glycemic control as he was titrated up to a dose of 4.2 mg/kg. After being treated for less than one week at this dose, the subject was withdrawn from the study due to worsening hyperglycemia. The subject was treated with insulin and returned to normoglycemia without need for further anti-diabetic therapy. Incidentally, at the time of withdrawal from the study, this subject had shown marked improvement in hyperphagia (-13, -62%). The investigator assessed this event to be related to study drug.

The safety profile of DCCR in PWS subjects was therefore consistent with prior studies of DCCR and with the historic safety profile of diazoxide. Most adverse events were mild to moderate and resolved while dosing continued. The impact on glucose was primarily transient. Peripheral edema, when it occurred, was often transient and responsive to management.

## Discussion

Diazoxide has been approved to treat hypoglycemia due to hyperinsulinemia in neonates, children and adults [16, 17]. The safety of long-term diazoxide treatment in hyperinsulinemia is well-known [18]. Children treated with the diazoxide remain on it for about 5 years, with an average dose of 12.5 mg/kg (equivalent to a diazoxide choline dose of about 18 mg/kg) [18], approximately 4 times greater than those used in this study of PWS patients. The historic use in the treatment of hypoglycemia due to hyperinsulinemia relies on agonization of the K_ATP_ channel at a distinct location–the beta cells of the pancreas [19].

The central action of DCCR should result in improvements in hyperphagia, hepatic and circulating lipids and in insulin sensitivity [6, 10–12]. The therapeutic responses observed in this pilot study of DCCR in PWS patients are consistent with this mode of action. Marked, statistically significant dose and baseline-severity dependent improvements in hyperphagia were observed. Concurrent treatment with growth hormone does not appear to have a detrimental effect on DCCR treatment. While hepatic lipids were not measured, there were marked improvements in circulating triglycerides, and statistically significant reductions in LDL-C and non-HDL-C.

Other central actions of DCCR related to amplifying GABA signaling in the context of low GABA and low GABA receptor numbers likely accounts for the improvement in aggressive and threatening behaviors [20–22]. Changes in lean body mass are likely due to the peripheral action of DCCR [23, 24].

The safety profile observed in this study is consistent with that observed in other clinical trials of DCCR and with the known safety profile of diazoxide [18].

Not surprisingly, with hyperphagia being the highest priority unmet need in PWS, other therapeutics have recently been evaluated for their effect on hyperphagia. Zafgen^™^ conducted a Phase III study of beloranib, a methionine aminopeptidase 2 inhibitor in PWS patients [25] which showed highly statistically significant improvements in hyperphagia and weight compared to placebo [25]. The study was terminated early however due to multiple thromboembolic events, two of which resulted in the death of treated patients [25]. Alize Pharma conducted a two weeks Phase II study of AZP-531, a once-daily injectable unacylated ghrelin analog, in primarily adult patients with PWS [26]. The study showed statistically significant effects on hyperphagia, which appear to be dependent on the care setting [26]. Intranasal oxytocin has been tested for its effects on hyperphagia in PWS in multiple studies, and statistical analysis showed a trend but no convincing evidence that intranasal oxytocin improves symptoms of PWS, including hyperphagia [27]. Intranasal carbetocin, an oxytocin analog has also been tested in PWS in a short (2-week) study and showed improvement in hyperphagia and some behavioral symptoms with an unusually high placebo response [28].

## Conclusions

Treatment of adolescent and adult, overweight and obese PWS patients with DCCR appeared to result in therapeutic benefits that followed from the mode of action. These improvements include statistically significant improvements in hyperphagia and its associated food-related behaviors in PWS patients, matching the highest priority unmet needs in the disease according to parents and caregivers. To the extent that this sample of PWS patients is reasonably representative of PWS patients in this age range, these results should apply to the broader PWS adolescent and young adult population. If comparable results are reported in the pivotal study of DCCR, the drug may be a preferred option to manage these high priority unmet needs of patients with PWS.

## Supporting information

S1 File Study protocol. (DOCX)

S2 File CONSORT checklist. (DOC)
