# Supplementary material for: A randomized pilot efficacy and safety trial of diazoxide choline controlled-release in patients with Prader-Willi syndrome
Source: PLoS One. 2019 Sep 23;14(9):e0221615. doi: 10.1371/journal.pone.0221615 (PMC6756513; doi:10.1371/journal.pone.0221615)
Supplement: S1 File — (DOCX) [file pone.0221615.s001.docx]

CLINICAL TRIAL PROTOCOL

(Phase 1)

**COMPOUND:** Diazoxide Choline

**TITLE**: A Dose Titration Study of
Diazoxide Choline Controlled-Release Tablet (DCCR) in
Patients with Prader-Willi syndrome with a Double-Blind,

Placebo-Controlled, Randomized Withdrawal Extension

**PROTOCOL NUMBER:** PC025

**VERSION: 4.5**

**VERSION DATE: May 23, 2014**

| **TRIAL MEDICAL MONITOR** | Function served by Data Safety Monitoring Board  Maria Cristina Kenney, MD, PhD, Professor  University of California, Irvine  John Jay Gargus, MD, PhD, Professor  University of California, Irvine  Madeleine Pahl, MD, Professor  University of California, Irvine |
| --- | --- |
| **PRINCIPAL INVESTIGATOR** | Name: Dr. Virginia Kimonis  Address: Dept. of Pediatrics, UC Irvine Division of Genetics and Metabolism PSF Division of Genetics Univ. of California-Irvine Med. Center 101 The City Drive South, ZC4482, Orange CA 92868  Phone: [(714) 456-5791](tel:%28714%29456-5791)  Fax: [(714) 456-5330](tel:%28714%29456-5330)  E-mail: vkimonis@uci.edu |

Table of Contents

[2. CLINICAL TRIAL PROTOCOL SYNOPSIS 7](#_Toc387910510)

[3. STUDY FLOW CHART 13](#_Toc387910511)

[4. ABBREVIATIONS and defined terms 14](#_Toc387910512)

[5. INTRODUCTION 15](#_Toc387910513)

[5.1 Background 15](#_Toc387910514)

[6. OBJECTIVES 17](#_Toc387910515)

[6.1 Primary Objectives 17](#_Toc387910516)

[6.2 Secondary Objectives 17](#_Toc387910517)

[6.3 Exploratory Objectives 17](#_Toc387910518)

[7. DESIGN 17](#_Toc387910519)

[8. POPULATION 18](#_Toc387910520)

[8.1 Number of Patients 18](#_Toc387910521)

[8.2 Inclusion Criteria 18](#_Toc387910522)

[8.3 Exclusion Criteria 18](#_Toc387910523)

[9. TREATMENT 19](#_Toc387910524)

[9.1 Investigational Drug Product 19](#_Toc387910525)

[9.1.1 Dose Selection 19](#_Toc387910526)

[9.1.2 Packaging and Labeling 21](#_Toc387910527)

[9.1.3 Responsibilities 21](#_Toc387910528)

[9.1.4 Storage Conditions 22](#_Toc387910529)

[9.1.5 Blinding Method 22](#_Toc387910530)

[9.1.6 Assignment Method 22](#_Toc387910531)

[9.1.7 Administration Method 22](#_Toc387910532)

[9.1.8 Administered Dose 22](#_Toc387910533)

[9.1.9 Dose Titration Decision 23](#_Toc387910534)

[9.1.10 Treatment of Overdose 24](#_Toc387910535)

[9.1.11 Accountability of Investigational Drug Product 24](#_Toc387910536)

[9.1.12 Destruction 24](#_Toc387910537)

[9.2 Restrictions 24](#_Toc387910538)

[9.2.1 Concomitant Medications 24](#_Toc387910539)

[9.2.2 Prohibited Medications 24](#_Toc387910540)

[9.2.3 Over-the-counter (OTC) Medications 25](#_Toc387910541)

[9.2.4 Herbal and Dietary Supplements 25](#_Toc387910542)

[9.2.5 Dietary 25](#_Toc387910543)

[9.3 Lifestyle Guidelines 25](#_Toc387910544)

[9.4 Study Duration and Study Days 25](#_Toc387910545)

[9.4.1 Screening Period: Day -28 to Day -1 25](#_Toc387910546)

[9.4.2 Baseline Period: Day 0 25](#_Toc387910547)

[9.4.3 Open Label Treatment Period: Day 0 to Day 69 25](#_Toc387910548)

[9.4.4 Double-Blind, Placebo-Controlled, Randomized Withdrawal Extension: Day 70 to Day 97 25](#_Toc387910549)

[10. STUDY ENDPOINTS 26](#_Toc387910550)

[10.1 Primary 26](#_Toc387910551)

[10.2 Secondary 26](#_Toc387910552)

[10.3 Exploratory 26](#_Toc387910553)

[10.4 Safety Endpoints 26](#_Toc387910554)

[10.4.1 Physical Examination 26](#_Toc387910555)

[10.4.2 Vital Signs 27](#_Toc387910556)

[10.4.3 Laboratory Tests 27](#_Toc387910557)

[10.4.4 Adverse Events 27](#_Toc387910558)

[10.5 Assessment Methods 27](#_Toc387910559)

[10.5.1 Clinical 27](#_Toc387910560)

[10.5.2 Questionnaires 29](#_Toc387910561)

[10.5.3 Biological 29](#_Toc387910562)

[11. VISITS 30](#_Toc387910563)

[11.1 General Overview 30](#_Toc387910564)

[11.2 Assessments 31](#_Toc387910565)

[11.2.1 Visit 1 (Day -28 to -14; Screening) 31](#_Toc387910566)

[11.2.2 Visit 2 (Day 0; Baseline; Start of Treatment) 31](#_Toc387910567)

[11.2.3 Visit 3 (Day 13) 32](#_Toc387910568)

[11.2.4 Visit 4 (Day 27) 32](#_Toc387910569)

[11.2.5 Visit 5 (Day 41) 33](#_Toc387910570)

[11.2.6 Visit 6 (Day 55) 33](#_Toc387910571)

[11.2.7 Visit 7 (Day 69; End of the Open Label Treatment Period) 33](#_Toc387910572)

[11.2.8 Visit 8 (Day 97; End of Study) 34](#_Toc387910573)

[12. SAFETY MONITORING AND REPORTING 35](#_Toc387910574)

[12.1 Adverse Events 35](#_Toc387910575)

[12.1.1 General Definitions 35](#_Toc387910576)

[12.1.2 Causality Assessment of AE 35](#_Toc387910577)

[12.1.3 Abnormalities of Clinical and Laboratory Assessments 35](#_Toc387910578)

[12.1.4 Treatment Discontinuation and Follow-Up 35](#_Toc387910579)

[12.1.5 Other Considerations 36](#_Toc387910580)

[12.2 Serious Adverse Events 36](#_Toc387910581)

[12.2.1 Definition of Serious Adverse Event (SAE) 36](#_Toc387910582)

[12.2.2 SAE Communication and Follow-Up 36](#_Toc387910583)

[12.3 Safety Instructions Specific to the Study 36](#_Toc387910584)

[12.3.1 Hyperglycemia 36](#_Toc387910585)

[12.3.2 Fluid Retention and Peripheral Edema 36](#_Toc387910586)

[12.3.3 Electrocardiographic Changes 37](#_Toc387910587)

[12.3.4 Elevation of Serum Uric Acid 37](#_Toc387910588)

[12.3.5 Consideration in Use with Concomitant Antihypertensive Agents 37](#_Toc387910589)

[12.3.6 Effects on Anticoagulants 37](#_Toc387910590)

[13. TREATMENT OR STUDY DISCONTINUATION 38](#_Toc387910591)

[13.1 Treatment Discontinuation 38](#_Toc387910592)

[13.1.1 Reasons 38](#_Toc387910593)

[13.1.2 Procedures 38](#_Toc387910594)

[13.2 Study Discontinuation 38](#_Toc387910595)

[13.2.1 Reasons 38](#_Toc387910596)

[13.2.2 Procedures 38](#_Toc387910597)

[14. STATISTICAL ANALYSIS 39](#_Toc387910598)

[14.1 Sample Size 39](#_Toc387910599)

[14.2 Analysis Populations 39](#_Toc387910600)

[14.2.1 Open Label Treatment Period Endpoint Evaluable Treatment Population 39](#_Toc387910601)

[14.2.2 Double-Blind, Placebo-Controlled, Randomized Withdrawal Extension Endpoint Evaluable Population 39](#_Toc387910602)

[14.2.3 Safety Population 39](#_Toc387910603)

[14.3 Sample Size and Power 39](#_Toc387910604)

[14.3.1 Statistical Hypotheses and Control of the False Positive Rate 39](#_Toc387910605)

[14.3.2 Assumptions for Sample Size and Power 39](#_Toc387910606)

[14.3.3 Power for the period from Baseline to Day 69 40](#_Toc387910607)

[14.3.4 Power for the period from Day 69 to Day 97 41](#_Toc387910608)

[14.4 Statistical Methods 41](#_Toc387910609)

[14.4.1 Descriptive Analysis 41](#_Toc387910610)

[14.4.2 Paired T-Tests 42](#_Toc387910611)

[14.4.3 ANOVA 42](#_Toc387910612)

[14.4.4 Fisher’s Exact Test 42](#_Toc387910613)

[14.5 Demographic and Baseline Characteristics 42](#_Toc387910614)

[14.5.1 Study Medication 42](#_Toc387910615)

[14.5.2 Concomitant Medications 42](#_Toc387910616)

[14.6 Study Endpoints 42](#_Toc387910617)

[14.6.1 Safety Endpoints 42](#_Toc387910618)

[14.7 Secondary Efficacy Endpoints 43](#_Toc387910619)

[14.8 Exploratory Efficacy Endpoints 43](#_Toc387910620)

[15. STUDY COMMITTEES 44](#_Toc387910621)

[15.1 Steering Committee 44](#_Toc387910622)

[15.2 Data and Safety Monitoring Board 44](#_Toc387910623)

[16. ETHICAL CONSIDERATIONS 44](#_Toc387910624)

[16.1 Principles 44](#_Toc387910625)

[16.2 Informed Consent 44](#_Toc387910626)

[16.3 Ethics Committee 44](#_Toc387910627)

[16.4 Confidentiality 45](#_Toc387910628)

[17. ADMINISTRATIVE AND REGULATORY PROCEDURES 45](#_Toc387910629)

[17.1 Laws 45](#_Toc387910630)

[17.2 Clinical Trials Registration 45](#_Toc387910631)

[17.3 Curriculum Vitae 45](#_Toc387910632)

[17.4 Record Retention 45](#_Toc387910633)

[17.5 Data Protection 45](#_Toc387910634)

[17.6 Insurance 45](#_Toc387910635)

[18. CLINICAL TRIAL PROTOCOL AMENDMENTS 45](#_Toc387910636)

[19. STUDY MONITORING 46](#_Toc387910637)

[19.1 Responsibilities of the Investigator 46](#_Toc387910638)

[20. Premature Discontinuation of the Study 46](#_Toc387910639)

[20.1 Decision by the Investigator 46](#_Toc387910640)

[21. PROPERTY RIGHTS -wording 46](#_Toc387910641)

[22. Communication and Publication of study Results 46](#_Toc387910642)

[23. Inspections and Audits 46](#_Toc387910643)

[24. References 46](#_Toc387910644)

[25. Appendices 47](#_Toc387910645)

[25.1 Laboratory for Pharmacokinetic Measurements 47](#_Toc387910646)

# CLINICAL TRIAL PROTOCOL SYNOPSIS

| TITLE  A Dose Titration Study of Diazoxide Choline Controlled-Release Tablet (DCCR) in  Patients with Prader-Willi syndrome With a Double-Blind, Placebo-Controlled, Randomized Withdrawal Extension |
| --- |
| OBJECTIVES  Primary   - To evaluate the safety of multiple dose levels of DCCR in obese PWS patients   Secondary   - To evaluate the magnitude of effect of DCCR on hyperphagia in responding obese PWS patients - To determine the magnitude of effect of DCCR on resting energy expenditure in responding obese PWS patients   **Exploratory**   - To determine whether the impact of DCCR on hyperphagia in obese PWS patients is dose dependent - To determine whether the impact of DCCR on resting energy expenditure in obese PWS patients is dose dependent - To determine the impact of DCCR on weight in obese PWS patients - To determine the impact of DCCR on waist circumference in obese PWS patients - To determine the impact of DCCR on BMI in obese PWS patients - To determine the impact of DCCR on lipids in obese PWS patients - To determine the impact of DCCR on body fat mass in obese PWS patients - To determine the impact of DCCR on respiratory quotient in obese PWS patients - To determine the impact of DCCR on ghrelin in obese PWS patients - To determine the impact of DCCR on leptin in obese PWS patients |
| STUDY DESIGN   - This is a single-center, open-label, single-arm study with a double-blind, placebo-controlled, randomized withdrawal extension. Patients are initiated on a DCCR dose of about 1.5 mg/kg (maximum starting dose of 145 mg) and are titrated every 14 days to about 2.4 mg/kg, 3.3 mg/kg, 4.2 mg/kg, and 5.1 mg/kg (maximum dose of 507.5 mg). These DCCR doses are equivalent to diazoxide doses of 1.03, 1.66, 2.28, 2.9, and 3.52 mg/kg. The administered dose will be as close to the mg/kg dosing as can be achieved by the available dose strengths of DCCR. Patients will be up-titrated at each visit at the discretion of the investigator. All patients will be randomized in the double-blind, placebo-controlled, randomized withdrawal extension. Any patient who showed an increase in resting energy expenditure and/or a reduction in hyperphagia from Baseline through Day 55 or Day 69 will be designated a responder, whereas all others will be designated non-responders. Patients will be randomized in a 1:1 ratio either to continue on active treatment at the dose they were treated with on Day 69 or to the placebo equivalent of that dose for an additional 4 weeks. Randomization will be stratified on responder/non-responder.   Schematic   \| **Screening (28 Days)*** \| **Open Label Treatment Period (70 days)** \| \| \| \| \| \| **Double-Blind, Placebo-Controlled, Randomized Withdrawal Extension**  **(28 days)** \| \| --- \| --- \| --- \| --- \| --- \| --- \| --- \| --- \| \| Day -28 to Day -1 \| \| Baseline  Day 0 \| Day 13 \| Day 27 \| Day 41 \| Day 55 \| Day 69 \| Day 97 \| \|  \| \| 1.5 mg/kg \| 2.4 mg/kg \| 3.3 mg/kg \| 4.2 mg/kg \| 5.1 mg/kg \| 5.1 mg/kg \| Day 69 dose or Placebo Equivalent \|   *All screening procedures must be completed by Day -14   \|  \|  \| \| --- \| --- \| |
| NUMBER OF PATIENTS  Up to 12 patients |
| COUNTRIES AND SITES  USA, 1 site |
| INCLUSION CRITERIA  Basic requirements  Ability to follow verbal and written instructions with or without assistance from caregiver  Informed consent form signed by the subject or their legal guardian  Completed the screening process between 2 and 4 weeks prior to Baseline Visit  General demographic characteristics   - Male and female patients 10 to 20 years of age, inclusive at the time of dosing - Genetically confirmed Prader-Willi syndrome - BMI exceeds the 95^th^ percentile of the age specific BMI value on the CDC BMI charts or percent body fat ≥ 35% (The body fat criteria will only be used if there were a measurement made in the last 12 months of the Screening Visit, and the patient has not lost weight since the measurement.  Under all other circumstances, the BMI criteria will apply.)   Generally healthy as documented by the medical history, physical examination, vital sign assessments, 12-lead electrocardiogram (ECG), and clinical laboratory assessments  Specific laboratory test results  fasting glucose ≤ 126 mg/dL  HbA1c ≤ 6.5 % |
| EXCLUSION CRITERIA  Medications: recent, current, anticipated  Administration of investigational drugs within 1 month prior to Screening Visit  Anticipated requirement for use of prohibited medications (see Section 9.2.2)  History of allergic reaction or significant intolerance to:  Diazoxide  Thiazides  Sulfonamides  Lifestyle and care changes  Anticipate transitions in their care from family home to group home or other similar potentially disruptive changes  Specific diagnoses, medical conditions and history  Known type I or III hyperlipidemia  Known type 1 DM  Positive urine pregnancy test (in women of child-bearing age)  Congestive heart failure or known compromised cardiac reserve  Cancer within the past 5 years (not applicable to successfully treated basal cell carcinoma and successfully treated *in situ* cervical cancer)  Any other clinically significant endocrine, cardiovascular, pulmonary, neurological, psychiatric, hepatic, gastrointestinal, hematological, renal, or dermatological disease interfering with the assessments of the investigational drug, according to the Investigator  Sitting systolic blood pressure > 160 mm Hg and/or sitting diastolic blood pressure  > 100 mm Hg at Screening Visit  Specific laboratory test results  alanine aminotransferase (ALT) or aspartate aminotransferase (AST) > 2 × ULN  Creatinine > 2 mg/dL  Proteinuria grade > 2+  Any relevant biochemical abnormality interfering with the assessments of the investigational drug product |
| INVESTIGATIONAL DRUG PRODUCT  Diazoxide Choline Controlled-Release Tablet (DCCR) in 2 strengths and Placebo equivalents:  DCCR 145 mg containing 145 mg diazoxide choline  DCCR 72.5 mg containing 72.5 mg diazoxide choline  Placebo equivalent to DCCR 145 mg  Placebo equivalent to DCCR 72.5 mg |
| TREATMENT ASSIGNMENT AND ADMINISTRATION  All patients will titrate from 1.5 mg/kg to 5.1 mg/kg (a maximum of 507.5 mg/day) at the discretion of the investigator during the Open-Label Treatment Period. The dose will be administered orally once daily in the morning with meal that includes solid food  During the Double-Blind, Placebo-Controlled, Randomized Withdrawal Extension all patients will be randomized in a 1:1 ratio to one of two treatments: either to continue on the dose they were receiving on Day 69 or to receive the placebo equivalent of the dose they were receiving on Day 69. Any patient who showed an increase in resting energy expenditure and/or a reduction in hyperphagia from Baseline through Day 41 or Day 69 will be designated a responder, whereas all others will be designated non-responders. Randomization will be stratified on responder/non-responder. |
| DIETARY AND LIFESTYLE GUIDELINES   - Patients should not alter any critical element of their dietary regimen, except as directed by the principal investigator. - During the study, patients will be instructed to continue, without change, their exercise and other lifestyle habits. |
| STUDY DURATION  Up to 126 days total  Screening period: up to 28 days  Baseline period: 1 days  Open Label Treatment period (including the Baseline visit which is also the first day of dosing): 70 days  Double-Blind, Placebo-Controlled, Randomized Withdrawal Extension period: 28 days |
| STUDY ENDPOINTS  Secondary   - Hyperphagia change from Day 69 through Day 97 using hyperphagia questionnaire - Resting energy expenditure expressed as percent RDA change from Day 69 through Day 97   **Exploratory**   - Hyperphagia change from Baseline through Day 69 using hyperphagia questionnaire - Resting energy expenditure expressed as percent RDA change from Baseline through Day 69 - Weight percent change from Baseline through Day 69 - Waist circumference change from Baseline through Day 69 - Body Mass Index (BMI) change from Baseline through Day 69 - Body fat mass (as measured by DEXA) change from Baseline through Day 69 - Respiratory quotient (RQ) change from Baseline through Day 69 - Ghrelin change from Baseline through Day 69 - Leptin change from Baseline through Day 69 - Triglyceride percent change from Baseline through Day 69 - Total cholesterol percent change from Baseline through Day 69 - LDL cholesterol percent change from Baseline through Day 69 - HDL cholesterol percent change from Baseline through Day 69 - Non-HDL cholesterol percent change from Baseline through Day 69 - Weight percent change from Day 69 through Day 97 - Waist circumference change from Day 69 through Day 97 - BMI change from Day 69 through Day 97 - RQ change from Day 69 through Day 97 - Ghrelin change from Day 69 through Day 97 - Leptin change from Day 69 through Day 97 - Triglyceride percent change from Day 69 through Day 97 - Total cholesterol percent change from Day 69 through Day 97 - LDL cholesterol percent change from Day 69 through Day 97 - HDL cholesterol percent change from Day 69 through Day 97 - Non-HDL cholesterol percent change from Day 69 through Day 97 |
| SAFETY ENDPOINTS  Safety assessments |
| **VISITS**  8 visits total   - Screening Visit (28 to 14 days prior to Baseline Visit (Day -28 to Day -14)) screening procedures can be done on one day or spread over multiple days. - Baseline Visit (Day 0, dosing begins) - During Open Label Treatment Period on Days 13, 27, 41, 55, and 69 - During Double-Blind, Placebo-Controlled, Randomized Withdrawal Extension on Day 97 |
| **STATISTICAL ANALYSIS**   - Descriptive statistics (number of observations, mean, standard deviation, median, minimum and maximum) for all secondary and exploratory endpoints for the whole population during the Open Label Treatment Period (Baseline through Day 69) and by treatment arm for the Double-Blind, Placebo-Controlled, Randomized Withdrawal Extension (Day 69 through Day 97) - Hyperphagia, resting energy expenditure expressed as percent RDA, waist circumference, BMI, RQ, Ghrelin, Leptin change from Baseline through Day 69, and weight, triglycerides, total cholesterol, LDL cholesterol, HDL cholesterol and non-HDL cholesterol percent change from Baseline through Day 69 will be analyzed by paired t-test - To determine the magnitude of effect of DCCR on hyperphagia and resting energy expenditure expressed as percent RDA, an ANOVA will be conducted comparing the treatment arm to placebo for hyperphagia change from Day 69 through Day 97 and for resting energy expenditure percent RDA change from Day 69 through Day 97. The ANOVA will be conducted on the entire population and on the ‘responder’ sub-group. Waist circumference, BMI, RQ, Ghrelin, Leptin change from Day 69 through Day 97, and weight, triglycerides, total cholesterol, LDL cholesterol, HDL cholesterol and non-HDL cholesterol percent change from Day 69 to Day 97 will be subjected to a similar ANOVA. - Individual patient graphs will be created to present a visual assessment of the relationship between dose titration and response on hyperphagia and resting energy expenditure. |
| **COMMITTEES**  Data Safety Monitoring Board (DMSB) |

# STUDY FLOW CHART

| **Period (duration)** | **Screening (28 days)*** | **Open Label Treatment (70 days)** | | | | | | **Double-Blind Placebo-Controlled Randomized Withdrawal Extension (28 Days)** |
| --- | --- | --- | --- | --- | --- | --- | --- | --- |
| **Visit Number** | **1** | **2**  **(Baseline)** | **3** | **4** | **5** | **6** | **7** | **8** |
| **Day Number** | **-28 to -1** | **0** | **13** | **27** | **41** | **55** | **69** | **97** |
| Informed consent | X |  |  |  |  |  |  |  |
| Demography, medical history | X |  |  |  |  |  |  |  |
| Physical examination, weight, waist circumference | X | X | X | X | X | X | X | X |
| Height | X |  |  |  |  |  |  |  |
| Vital signs (heart rate, bp, temp) | X | X | X | X | X | X | X | X |
| ECG (12-lead) | X |  |  |  |  |  |  |  |
| DEXA, urine pregnancy test |  | X |  |  |  |  | X |  |
| Behavioral questionnaire |  | X |  |  |  |  | X |  |
| Urinalysis. LFTs, IGF-1 and HbA1c | X |  |  |  |  |  | X | X |
| Fasting glucose | X |  | X | X | X | X |  |  |
| 2 hour oral glucose tolerance test |  | X |  |  |  |  | X | X |
| Ghrelin, leptin, triglycerides, Total C, LDL-C, HDL-C, non-HDL-C, comprehensive metabolic panel |  | X |  |  |  |  | X | X |
| Hyperphagia questionnaire | X | X | X | X | X |  | X | X |
| Resting energy expenditure, respiratory quotient |  | X |  | X |  |  | X | X |
| Pharmacokinetic blood sampling |  |  |  |  |  |  | X | X |
| Enrollment |  | X |  |  |  |  |  |  |
| Medication accountability |  | X | X | X | X | X | X | X |
| Medication dispensation |  | X | X | X | X | X | X |  |
| Randomization in Extension |  |  |  |  |  |  | X |  |
| Adverse events | X | X | X | X | X | X | X | X |

*All screening procedures must be completed by Day -14

# ABBREVIATIONS and defined terms

**Table 1. List of Abbreviations**

| **Abbreviation** | **Terminology** |
| --- | --- |
| AE | adverse event |
| ALP | alkaline phosphatase |
| ALT | alanine aminotransferase |
| am | ante meridiem |
| AST | aspartate aminotransferase |
| ATP | adenosine triphosphate |
| bp | blood pressure |
| BMI | body mass index |
| C | Celsius |
| CDC | US Center for Disease Control |
| CRF | case report form |
| DBP | diastolic blood pressure |
| DCCR | Diazoxide Choline Controlled-Release Tablet |
| DEXA | Dual-Energy X-Ray Absorptiometry |
| dL | deciliter |
| DM | diabetes mellitus |
| DRF | discrepancy resolution form |
| ECG | electrocardiogram |
| F | Fahrenheit |
| FDA | Food and Drug Administration |
| GCP | Good Clinical Practices |
| h | hour |
| HbA1c | glycosylated hemoglobin |
| HDL-C | high density lipoprotein cholesterol |
| ICH | International Conference on Harmonization |
| IGF-1 | Insulin-like growth factor-1 |
| IRB | Institutional Review Board |
| IV | intravenous |
| kg | kilogram |
| L | liter |
| LDL-C | low density lipoprotein cholesterol |
| M.D. | Medical Doctor |
| mg | milligram |
| min | minute |
| mL | milliliter |
| mm Hg | millimeters of mercury |
| μg | microgram |
| NOAEL | no observed adverse effect level |
| Non-HDL-C | non-high density lipoprotein cholesterol |
| PK | pharmacokinetics or pharmacokinetic |
| PO | per os, Latin for by mouth (oral) |
| PWS | Prader-Willi syndrome |
| QD | quaque die, Latin for once a day |
| RDA | recommended daily allowance |
| RQ | respiratory quotient |
| SAE | serious adverse event |
| SAP | statistical analysis plan |
| SBP | systolic blood pressure |
| temp | temperature |
| Total-C | total cholesterol |
| TSH | thyroid stimulating hormone (thyrotropin) |
| ULN | upper limit of normal |
| US | United States |
| USA | United States of America |
| WHO | World Health Organization |

# INTRODUCTION

## Background

Diazoxide choline controlled-release tablet (DCCR) is a patent protected, QD tablet formulation of diazoxide choline. Diazoxide choline is a novel, highly crystalline salt of diazoxide. The molecular weight of diazoxide choline is 1.45 times that of diazoxide. Thus, every 145 mg of diazoxide choline contains the equivalence of 100 mg diazoxide.

Upon administration of DCCR, prior to absorption, diazoxide choline hydrolyzes to diazoxide. Diazoxide is a benzothiadiazine that acts by stimulating ion flux through the ATP sensitive potassium channel (K_ATP_ channel). Under normal physiologic conditions the K_ATP_ channel is antagonized by intracellular ATP and activated by intracellular nucleoside diphosphates.

Nearly 40 years ago, diazoxide was approved in two formulations in the US for two key indications. It has been approved by the FDA as an oral suspension for the treatment of a range of hyperinsulinemic hypoglycemic conditions. It had also been approved by the FDA as an intravenous (IV) solution for the emergency treatment of malignant hypertension. There has been a long history of safe use of oral formulations of diazoxide in the treatment of hyperinsulinemic hypoglycemic conditions in neonates, children and adults.

Data from animal models and human studies show that diazoxide has the potential to provide therapeutic benefit to patients with Prader-Willi Syndrome. The underlying hypothesis for development of diazoxide choline in this indication is that agonizing the K_ATP_ channel in hypothalamic neurons will reduce appetite and increase energy expenditure. This effect is complemented by the peripheral effect of diazoxide on adipocytes where agonizing the K_ATP_ channel reduces fatty acid biosynthesis and increases β-oxidation of fat, thereby reducing fat stores. The overall impact of the hypothalamic and peripheral effects of diazoxide in PWS patients is anticipated to result in reductions in hyperphagia, increases in resting energy expenditure, a switch from burning carbohydrate as fuel to burning fat as fuel and a reduction in the rate of weight gain. These effects of diazoxide are anticipated to be observed in the dose range selected for use in this clinical study. All of the doses tested in this clinical study have been shown to be well tolerated in other pediatric and adult patient populations when administered either as diazoxide or DCCR.

A review of the most recent 20 years of treatment experience with diazoxide in pediatric patients was conducted, including more than 90 peer reviewed publications describing the treatment of more than 2,600 pediatric patients. Use of diazoxide in these patients first involves a trial treatment period to determine diazoxide responsiveness which is typically conducted using a dose of 15 mg/kg. Thereafter the dose is adjusted up or down to a maintenance dose. Hyperinsulinemic/hyperammonemia patients can be effectively treated with doses of 3-5 mg/kg along with a carefully controlled diet. Most infant onset chronic hypoglycemia of infancy (CHI) can be controlled using doses between 5 and 15 mg/kg, while neonatal onset CHI tends to be treated with doses of 10 to 20 mg/kg. Treatment at these doses continues for years. The average dose in these treated pediatric patients is between 10 and 15 mg/kg which is 2.8x to 4.3x the highest dose tested in this study.

The most common adverse event (AE) associated with diazoxide treatment of pediatric patients is fluid retention, which is diuretic responsive. It is dose dependent in its incidence and severity.

Diazoxide is intrinsically diuretic and natriuretic. Diazoxide is also associated with increased glomular filtration rate and renal plasma flow. The etiology of diazoxide induced peripheral edema relates to a decrease in arteriolar resistance that is unmatched in the venous or postcapillary circulation. The disproportionate change in resistance increases hydrostatic pressures in the precapillary circulation and forces fluid shifts into the interstitial compartment. It appears that this effect is most pronounced at the initiation of therapy. Initially, this transcapillary fluid movement exceeds the capacity of the lymphatic system to clear it resulting in clinically evident edema. There is clear evidence from the literature and from Essentialis clinical experience that diazoxide induced peripheral edema is transient. This etiology is indistinguishable from the etiology of fluid retention and edema associated with calcium channel blockers.

The second most common adverse event associated with diazoxide treatment of pediatric patients is hirsutism. The hirsutism is associated with the agonizing of the K_ATP_ channel in the hair follicle. Hirsutism is more common in young children than older children and is dose dependent in its incidence and extent. Other K_ATP_ channel agonists are approved to treat hair loss (minoxidil – Rogaine). The condition resolves following the cessation of treatment, or when dose is reduced.

There have been a number of cases of rash, allergic or anaphylactic reactions to diazoxide. In clinical studies with DCCR, it appears that these occur in individuals who also have sulfa drug allergies. Diazoxide includes a sulfur atom its structure which may contribute to these reactions.

Headaches, nausea and vomiting may occur at the initiation of therapy with diazoxide, particularly if the starting dose is high, or the dose is titrated aggressively.

Rarely, there have been cases of thrombocytopenia, pancytopenia or leukopenia reported with diazoxide. Nearly all of these occurred at high doses of diazoxide when a thiazide diuretic was co-administered. Thrombocytopenia, leukopenia, agranulocytosis, and hemolytic anemia are well-characterized adverse effects of thiazide diuretics. The combination of diazoxide and thiazide diuretics at higher doses is known to suppress bone marrow in animal studies.

# OBJECTIVES

This Phase 1 study has the following objectives.

## Primary Objectives

- To evaluate the safety of multiple dose levels of DCCR in obese PWS patients

## Secondary Objectives

- To evaluate the magnitude of effect of DCCR on hyperphagia in responding obese PWS patients
- To determine the magnitude of effect of DCCR on resting energy expenditure in responding obese PWS patients

## Exploratory Objectives

- To determine whether the impact of DCCR on hyperphagia in obese PWS patients is dose dependent
- To determine whether the impact of DCCR on resting energy expenditure in obese PWS patients is dose dependent
- To determine the impact of DCCR on weight in obese PWS patients
- To determine the impact of DCCR on waist circumference in obese PWS patients
- To determine the impact of DCCR on BMI in obese PWS patients
- To determine the impact of DCCR on lipids in obese PWS patients
- To determine the impact of DCCR on body fat mass in obese PWS patients
- To determine the impact of DCCR on respiratory quotient in obese PWS patients
- To determine the impact of DCCR on ghrelin in obese PWS patients
- To determine the impact of DCCR on leptin in obese PWS patients

# DESIGN

- This is a single-center, open-label, single-arm study with a double-blind, placebo-controlled, randomized withdrawal extension. Patients are initiated on a DCCR dose of about 1.5 mg/kg (maximum starting dose of 145 mg) and are titrated every 14 days to about 2.4 mg/kg, 3.3 mg/kg, 4.2 mg/kg, and 5.1 mg/kg (maximum dose of 507.5 mg). These DCCR doses are equivalent to diazoxide doses of 1.03, 1.66, 2.28, 2.9, and 3.52 mg/kg. The administered dose will be as close to the mg/kg dosing as can be achieved by the available dose strengths of DCCR. Patients will be up-titrated at each visit at the discretion of the investigator. All patients will be randomized in the double-blind, placebo-controlled, randomized withdrawal extension. Any patient who showed an increase in resting energy expenditure and/or a reduction in hyperphagia from Baseline through Day 41 or Day 69 will be designated a responder, whereas all others will be designated non-responders. Patients will be randomized in a 1:1 ratio either to continue on active treatment at the dose they were treated with on Day 69 or to the placebo equivalent of that dose for an additional 4 weeks. Randomization will be stratified on responder/non-responder.

Schematic

| **Screening (28 Days)*** | **Open Label Treatment Period (70 days)** | | | | | | **Double-Blind, Placebo-Controlled, Randomized Withdrawal Extension**  **(28 days)** |  |
| --- | --- | --- | --- | --- | --- | --- | --- | --- |
| Day -28 to Day -1 | | Baseline  Day 0 | Day 13 | Day 27 | Day 41 | Day 55 | Day 69 | Day 97 |
|  | | 1.5 mg/kg | 2.4 mg/kg | 3.3 mg/kg | 4.2 mg/kg | 5.1 mg/kg | 5.1 mg/kg | Day 69 dose or Placebo Equivalent |

*All screening procedures must be completed by Day -14

# POPULATION

The population will consist of patients with genetically confirmed Prader-Willi syndrome.

## Number of Patients

Up to 12 patients will be enrolled in the study at 1 site in the USA.

## Inclusion Criteria

Basic requirements

1. Ability to follow verbal and written instructions with or without assistance from caregiver
2. Informed consent form signed by the subject or their legal guardian
3. Completed the screening process within 2 and 4 weeks prior to Baseline Visit 1

General demographic characteristics

1. Male and female patients 10 to 20 years of age, inclusive at the time of dosing
2. Genetically confirmed Prader-Willi syndrome
3. BMI exceeds the 95^th^ percentile of the age specific BMI value on the CDC BMI charts or percent body fat ≥ 35% (The body fat criteria will only be used if there were a measurement made in the last 12 months of the Screening Visit, and the patient has not lost weight since the measurement.  Under all other circumstances, the BMI criteria will apply.)
4. Generally healthy as documented by the medical history, physical examination, vital sign assessments, 12-lead electrocardiogram (ECG), and clinical laboratory assessments

Specific laboratory test results

1. Fasting glucose ≤ 126 mg/dL
2. HbA1c ≤ 6.5 %

## Exclusion Criteria

Medications: recent, current, anticipated

1. Administration of investigational drugs within 1 month prior to Screening Visit
2. Anticipated requirement for use of prohibited medications (see Section 9.2.2)

History of allergic reaction or significant intolerance to:

1. Diazoxide
2. Thiazides
3. Sulfonamides

Lifestyle changes

1. Anticipated transition in their care from family home to group home or other similar potentially disruptive changes

Specific diagnoses, medical conditions and history

1. Known type I or III hyperlipidemia
2. Known type 1 DM
3. Positive urine pregnancy test (in women of child-bearing age)
4. Congestive heart failure or known compromised cardiac reserve
5. Cancer within the past 5 years (not applicable to successfully treated basal cell carcinoma and successfully treated in situ cervical cancer)
6. Any other clinically significant endocrine, cardiovascular, pulmonary, neurological, psychiatric, hepatic, gastrointestinal, hematological, renal, or dermatological disease interfering with the assessments of the investigational drug, according to the Investigator
7. Sitting systolic blood pressure > 160 mm Hg and/or sitting diastolic blood pressure
   > 100 mm Hg at the Screening Visit)

Specific laboratory test results

1. alanine aminotransferase (ALT) or aspartate aminotransferase (AST) > 2 × ULN
2. Creatinine > 2 mg/dL
3. Proteinuria grade > 2+
4. Any relevant biochemical abnormality interfering with the assessments of the investigational drug

# TREATMENT

## Investigational Drug Product

As described in Section 5.1, DCCR is a patented QD tablet formulation of diazoxide choline.

DCCR 145 mg, containing 145 mg diazoxide choline, and DCCR 72.5 mg, containing 72.5 mg diazoxide choline, will be the investigational drug products used to achieve the DCCR dose levels targeted during the Open Label Treatment Period.

DCCR 145 mg, DCCR 72.5 mg, and placebo equivalents to DCCR 145 mg and DCCR 72.5 mg will be the investigational drug products used to achieve the targeted dose level during the Double-Blind, Placebo-Controlled, Randomized Withdrawal Extension of the study.

### Dose Selection

The labeled dosing of diazoxide (Proglycem^®^ oral suspension) in adults is 3 to 8 mg/kg/day. The labeled dosing of diazoxide in neonates and children is 8 to 15 mg/kg. The maximal clinical dose of diazoxide tested in adults is greater than 20 mg/kg/day. Neonates and children are routinely treated at doses of 20 mg/kg for years. There are case reports of obese subjects receiving 2,200 mg/day (9). In rats, the maximum tolerated dose of diazoxide, administered as diazoxide choline in a single dose study, is 500 mg/kg, and the NOAEL (no observed adverse effect level) of diazoxide, administered as diazoxide choline in a 90-day repeat dose study, is 60 mg/kg/day.

The molecular weight of diazoxide choline is 1.45 times that of diazoxide. Thus, every 145 mg of diazoxide choline contains the equivalent of 100 mg diazoxide. The administered doses in this study, 1.5 mg/kg (maximum starting dose of 145 mg), 2.4 mg/kg, 3.3 mg/kg, 4.2 mg/kg and 5.1 mg/kg are equivalent to diazoxide doses of 1.03, 1.66, 2.28, 2.9 and 3.52 mg/kg.

All doses in this dose range have been shown to be well tolerated in other pediatric and adult patient populations when administered either as diazoxide or DCCR. Based upon a review of all published literature from the last 20 years covering the use of diazoxide in children (93 publications describing treatment of more than 2500 children), it appears that the average patient treated chronically with diazoxide receives a dose between 10 and 15 mg/kg. Treatment at these doses frequently continues for years, with the longest duration described in the reviewed literature being 22 years. Recall that diazoxide doses of 10 and 15 mg/kg are equivalent to DCCR doses of 14.5 and 21.75 mg/kg.

Results from an Essentialis Phase 1 trial, Study TR002, using multiple doses of DCCR (145 mg/day to 580 mg/day for 14 days to 49 days, with or without titration), administered to obese patients on a reduced calorie diet showed weight loss in excess of diet controls at doses of 290 mg and above. Titration of DCCR from 217.5 mg/day to 507.5 mg/day in obese patients on a reduced calorie diet showed that the drug was well tolerated over this dose range.

Results from an Essentialis Phase 2 trial, Study PC007 using multiple dose levels of DCCR (290 mg/day to 580 mg/day) administered to hypertriglyceridemic patients show that all doses in this range were well tolerated.

Alemzadeh et al. (5) evaluated the impact of 8 weeks of diazoxide treatment on weight loss in morbidly obese subjects. All subjects were placed on a low-calorie Optifast diet (1260 kcal for females and 1570 kcal for males). After an initial 1-week dietary lead-in period, 12 subjects received diazoxide (2 mg/kg/day; maximum 200 mg/day, effective dose of 1.8 mg/kg/day, divided into 3 doses) for 8 weeks; and 12 subjects received placebo. Compared with the placebo group, diazoxide treated subjects had greater weight loss (9.5±0.7% of body mass *vs.* 4.6±0.6%), greater decrease in body fat, and greater increase in fat-free mass to body fat ratio than did the placebo treated arm. HbA_1c_ values were reduced by diazoxide treatment but rose incrementally from pretreatment values in the placebo arm. Virtually all of the weight loss in the treatment arm was attributable to loss of body fat (weight loss: 9.8 kg, body fat loss: 9.3 kg).

Van Boekel et al. (6) conducted an open label, uncontrolled 6-month study of diazoxide in obese men. Dosing was initiated at 150 mg/day in 3 divided doses and increased every 4 weeks by 50 150 mg/day to a maximum of 900 mg/day in 3 divided doses. Subjects were titrated to intolerability and then dose reduced by 150 mg/day. Weight loss was evident at the starting dose (i.e. 150 mg/day). The average diazoxide dose during the latter half of the study was 600 mg/day. During the treatment period, body weight decreased by 9.4 kg (95% CI: 5.6–13.2 kg, p < 0.001), waist circumference decreased by 9.2 cm (95% CI: 5.3–12.9 cm, p < 0.001) and total body fat mass decreased by 23.3% (95% CI: 13.7–32.9%, p < 0.001), without a concomitant change in soft tissue lean body mass or bone mass. Virtually all of the weight loss was attributable to loss of body fat as measured by DEXA. Given the weight loss, it is not surprising that HDL rose by 11%, LDL dropped by 7% and triglycerides were down by 50%. There was a reduction in diastolic blood pressure of about 10 mmHg without a change in systolic pressure. Diazoxide as a vasodilator has a greater impact on diastolic than systolic pressure.

Hamilton et al. (7) assessed the effect of combined diazoxide-metformin therapy in obese adolescents (average age 15) surgically treated for craniopharyngioma following which they presented with hypothalamic obesity, in a prospective, open-label, 6-month pilot treatment trial in 9 obese adolescent patients. Subjects were followed for 6 months prior to treatment. Diazoxide (2 mg/kg divided into two equal doses, maximum 200 mg/day) and metformin (1000 mg twice daily) were administered for an additional 6 months. Seven subjects completed the treatment period, 4 males and 3 females. Their baseline average weight and BMI were 99.7 kg and 35.5 kg/m^2^, respectively. Two were withdrawn, one due to vomiting (likely an effect of metformin) and one due to peripheral edema (likely an effect of diazoxide). Of participants completing the study, the mean ± SD weight gain and BMI change during the 6 months were reduced compared to the 6 months prestudy (+1.2 ± 5.9 versus +9.5 ± 2.7 kg, P = .004; −0.3 ± 2.3 versus +2.2 ± 1.5 kg/m^2^, p = .04). The authors concluded that combined diazoxide-metformin therapy was associated with reduced weight gain in patients with hypothalamic obesity.

Young PWS patients, prior to presenting with hyperphagia, showed increases in resting energy expenditure and other beneficial effects at diazoxide doses of 2.0 to 2.5 mg/kg.

### Packaging and Labeling

The Investigator will have adequate investigational drug product, in bulk supply. The batch numbers for the drug product and packaged drug product will be provided to the study site on the packing slip with the product shipment. The investigational drug product will be transferred from bulk supplies into the subject’s individual dose administration container by qualified pharmacy staff while ensuring minimum temperature change during the transfer. Each unit dose administration container will be appropriately labeled in accordance with the regulatory specifications and requirements, with content information, dosing instructions, and precautionary statement (“for clinical use only”, “keep out of reach of children”). In addition the label for the dose administration container will specify Subject Number, and Protocol/Study number.

### Responsibilities

The Investigator (or designee) will maintain an accurate record of receipt of the investigational drug product as shipped, including the date received.

The personnel in charge of the storage and dispensation of the investigational drug product will insure that the product is securely maintained as specified by the Sponsor and in accordance with the regulatory requirements.

The investigational drug product will be used for the purpose of this study only, and will be dispensed in accordance with the Investigator’s prescription. It is the responsibility of the Investigator to ensure that an accurate record of investigational drug product dispensed and returned is maintained. The drug disposition record will specify the amount dispensed to each subject and the date of dispensation. The inventory record will be available for inspection at any time.

### Storage Conditions

The investigational drug product will be sent to the Investigator (according to local or federal regulatory requirements). It will be stored at the study site, in a secure and safe place, between 15°C and 30°C (59°F and 86°F), under the responsibility of the Investigator or other authorized individual.

Patients will be given the same instructions on storage conditions for storing the investigational drug product at home.

### Blinding Method

The patient, the clinical site personnel involved in the study (except the pharmacy staff), and the Sponsor will be blinded from the treatment assigned to the subject during the Double-Blind, Placebo-Controlled, Randomized Withdrawal Extension. Placebo tablets matching the size, shape, color, and coating of each of the dose strengths of DCCR will be used for blinding the study treatment.

### Assignment Method

All patients will titrate from 1.5 mg/kg to a maximum of 5.1 mg/kg (or 507.5 mg/day) during the Open-Label Treatment Period. The dose will be administered orally once daily in the morning with meal that includes solid food.

During the Double-Blind, Placebo-Controlled, Randomized Withdrawal Extension all patients with be randomized in a 1:1 ratio to one of two treatments: either to continue on the dose they were receiving on Day 69 or to receive the placebo equivalent of the dose they were receiving on Day 69. Randomization will be stratified on responder/non-responder.

### Administration Method

The DCCR dose is to be administered by mouth (per os or PO) once a day (quaque die or QD) in the morning (preferably before 9 am) at approximately the same time, with 240 mL of room temperature water, and within 15 min of food consumption that includes solid food.

The purpose of dosing DCCR with solid food is to help minimize potential GI-related adverse events. Therefore patients are not required to consume a certain percentage of the meal provided or a certain amount of food prior to dosing at home.

### Administered Dose

The numbers of tablets of each dose strength of DCCR required to achieve the target dose for patient weights between 30 and 119 kg are presented in Table 2.

Table 2 Numbers of Tablets of Each Dose Strength Required to Achieve Dose

|  |  | **Patient weight rounded to nearest kg** | | | | | | | | |
| --- | --- | --- | --- | --- | --- | --- | --- | --- | --- | --- |
| **Dose** | **Dose strengths** | **30 to 39 kg** | **40 to 49 kg** | **50 to 59 kg** | **60 to 69 kg** | **70 to 79 kg** | **80 to 89 kg** | **90 to 99 kg** | **100 to 109 kg** | **110 to 119 kg** |
| **1.5 mg/kg** | **145 mg tablets** | 0 | 0 | 0 | 0 | 0 | 1 | 1 | 1 | 1 |
|  | **72.5 mg tablets** | 1 | 1 | 1 | 1 / 2 | 1 / 2 | 0 | 0 | 0 | 0 |
| **2.4 mg/kg** | **145 mg tablets** | 0 | 0 | 1 | 1 | 1 | 1 | 1 | 1 | 1 |
|  | **72.5 mg tablets** | 1 | 1 / 2 | 0 | 0 | 0 / 1 | 0 / 1 | 1 | 1 / 2 | 1 / 2 |
| **3.3 mg/kg** | **145 mg tablets** | 0 | 1 | 1 | 1 | 1 | 2 | 2 | 2 | 2 |
|  | **72.5 mg tablets** | 1 / 2 | 0 | 0 / 1 | 1 | 1 / 2 | 0 | 0 | 0 / 1 | 1 |
| **4.2 mg/kg** | **145 mg tablets** | 1 | 1 | 1 | 2 | 2 | 2 | 2 | 3 | 3 |
|  | **72.5 mg tablets** | 0 | 0 / 1 | 1 | 0 | 0 / 1 | 1 | 1 / 2 | 0 | 0 / 1 |
| **5.1 mg/kg** | **145 mg tablets** | 1 | 1 | 2 | 2 | 2 | 3 | 3 | 3 | 3 |
|  | **72.5 mg tablets** | 0 / 1 | 1 | 0 | 0 / 1 | 1 | 0 | 0 / 1 | 1 | 1 |

Table 2 includes eighteen combinations of Dose and Patient weight where the numbers of 72.5 mg tablets are listed either as 0 / 1 or 1 / 2. These refer to situations in which every other day dosing with one 72.5 mg tablet is used to achieve the target dose. To achieve the dose for each of these 18 combinations of Dose and Patient weight, in addition to administering the indicated numbers of 145 mg tablets, patients will, on consecutive days of dosing, receive either zero or one (identified in Table 2 as 0 / 1) 72.5 mg tablets or one or two (identified in Table 2 as 1 / 2) 72.5 mg tablets. By way of example, a 70 kg patient receiving the 2.4 mg Dose on the first, third, fifth, seventh, ninth, eleventh and thirteenth day of dosing in the 14 day dosing period at the 2.4 mg Dose, before the next titration step, would receive one 145 mg tablet and zero 72.5 mg tablets. This patient on the second, fourth, sixth, eighth, tenth, twelfth, and fourteenth day of dosing in the 14 day dosing period at the 2.4 mg Dose, before the next titration step, would receive one 145 mg tablet and one 72.5 mg tablet. Similarly, a 90 kg patient receiving the 4.2 mg Dose on the first, third, fifth, seventh, ninth, eleventh and thirteenth day of dosing in the 14 day dosing period at the 4.2 mg Dose, before the next titration step, would receive two 145 mg tablets and one 72.5 mg tablet. This patient on the second, fourth, sixth, eighth, tenth, twelfth, and fourteenth day of dosing in the 14 day dosing period at the 4.2 mg Dose, before the next titration step, would receive two 145 mg tablets and two 72.5 mg tablets.

### Dose Titration Decision

At the Visits on Days 13, 27, 41, and 55 the patient should be up-titrated on dose to 2.4 mg/kg, 3.3 mg/kg, 4.2 mg/kg, and 5.1 mg/kg, respectively. The Investigator, at their discretion, may choose to maintain the patient on the administered dose, or may reduce the dose. The decision to maintain the patient on the administered dose, or to dose reduce, should only be made on the basis of the patient’s tolerance of the administered dose or anticipated ability to tolerate the next higher dose. Titration of dose should occur even if a patient has a clinical relevant response to treatment based on hyperphagia, resting energy expenditure or other efficacy parameter. Some AEs which may limit the ability to continue to titrate the dose include allergic reactions to DCCR, marked fluid retention or peripheral edema, marked increases in fasting glucose, headaches, or nausea and vomiting. For example, if a patient is experiencing an adverse event that is anticipated to be transient and resolve while dosing continues, the Investigator may choose to maintain the dose rather than up-titrate the dose. Similarly, if a patient is experiencing an adverse event that is not expected to be transient if the dose is continued and which might otherwise lead to a treatment discontinuation, the Investigator may choose to reduce the dose. Having made a decision to maintain dose, or dose reduce, at one Visit, the Investigator may, at their discretion, continue titration at the subsequent Visit.

### Treatment of Overdose

Overdosage with diazoxide choline (from DCCR) may cause severe hyperglycemia, fluid retention, and/or undesirable hypotension. Hyperglycemia is best treated by insulin administration. Fluid retention can be treated with diuretics. Hypotension usually responds to sympathomimetic agents. Because of the long half-life of diazoxide (29-32 hrs), according to the Proglycem label, peritoneal dialysis or hemodialysis may also be proposed in some cases. The Investigator is responsible for any decision regarding the treatment of overdose.

### Accountability of Investigational Drug Product

Usage of the investigational drug product will be assessed by counting of tablets remaining in each unit dose container, at each visit.

Accountability of the investigational drug product will be under Investigator’s direct supervision. The appropriate case report form (CRF) pages will be completed regarding the dosing and the treatment received.

### Destruction

At the completion of the study, investigational drug product, including all empty, partially used, and unused treatments, will be destroyed. A detailed treatment log will be established and countersigned by the Investigator and the Monitoring Team Representative (or designee).

## Restrictions

Patients who violate any of the following restrictions may be excluded or dropped from the study at the discretion of the investigator(s). Individual exceptions to the following restrictions may be approved by Sponsor’s Representative.

### Concomitant Medications

Use of recombinant human growth hormone is allowed. Stable doses of prescription medications for mild, chronic conditions are allowed at the investigator’s discretion. If, for medical reasons, any non-investigational drug product is administered during the study, the investigator will record all pertinent information.

### Prohibited Medications

The following medications are prohibited throughout the study:

Anti-diabetic medications except metformin

Anti-obesity medications or other medications (including herbal preparations) for weight reduction

### Over-the-counter (OTC) Medications

Patients should refrain from:

Use of any over-the-counter medications 3 days prior to dosing and through the end of the study.

### Herbal and Dietary Supplements

Patients should not have:

used any herbal or dietary supplements for a period of 14 days prior to dosing and throughout the study

used a therapeutic dose of any vitamins for a period of 3 days prior to dosing and throughout the study.

### Dietary

Patients should not alter any critical element of their dietary regimen, from the Screening Visit through the End of the Study, except as directed by the principal investigator.

## Lifestyle Guidelines

During the study, patients will be instructed to continue, without change, their exercise and other lifestyle habits.

## Study Duration and Study Days

The duration of the entire study is up to 126 days, with intervals as defined below.

### Screening Period: Day -28 to Day -1

The Screening Period is defined by the interval from Day - 28 to Day -1, i.e., from 42 days to 1 day prior to the Baseline Visit on Day 0. During the 28-day Screening Period, the Screening procedures must be done within the interval from Day -28 to Day -14 and can be all done on one day or spread over multiple days. Thus, all screening procedures must be completed by Day -14.

### Baseline Period: Day 0

There will be a Baseline Visit on Day 0.

### Open Label Treatment Period: Day 0 to Day 69

Day 0, the day of the Baseline assessment, is also the day the study treatment begins after completion of Baseline assessment.

The duration of the Open Label Treatment Period is 70 days.

### Double-Blind, Placebo-Controlled, Randomized Withdrawal Extension: Day 70 to Day 97

The duration of the Double-Blind, Placebo-Controlled, Randomized Withdrawal Extension is 28 days.

# STUDY ENDPOINTS

## Primary

- The primary objective is to evaluate safety. The safety endpoints, including adverse events and laboratory measurements, are to be considered co-primary endpoints.

## Secondary

- Hyperphagia change from Day 69 through Day 97 using hyperphagia questionnaire
- Resting energy expenditure expressed as percent RDA change from Day 69 through Day 97

## Exploratory

- Hyperphagia change from Baseline through Day 69 using hyperphagia questionnaire
- Resting energy expenditure expressed as percent RDA change from Baseline through Day 69
- Weight change from Baseline through Day 69
- Waist circumference change from Baseline through Day 69
- Body Mass Index (BMI) change from Baseline through Day 69
- Body fat mass (as measured by DEXA) change from Baseline through Day 69
- Respiratory quotient (RQ) change from Baseline through Day 69
- Ghrelin change from Baseline through Day 69
- Leptin change from Baseline through Day 69
- Triglyceride percent change from Baseline through Day 69
- Total cholesterol percent change from Baseline through Day 69
- LDL cholesterol percent change from Baseline through Day 69
- HDL cholesterol percent change from Baseline through Day 69
- Non-HDL cholesterol percent change from Baseline through Day 69
- Weight change from Day 69 through Day 97
- Waist circumference change from Day 69 through Day 97
- BMI change from Day 69 through Day 97
- RQ change from Day 69 through Day 97
- Ghrelin change from Day 69 through Day 97
- Leptin change from Day 69 through Day 97
- Triglyceride percent change from Day 69 through Day 97
- Total cholesterol percent change from Day 69 through Day 97
- LDL cholesterol percent change from Day 69 through Day 97
- HDL cholesterol percent change from Day 69 through Day 97
- Non-HDL cholesterol percent change from Day 69 through Day 97

## Safety Endpoints

### Physical Examination

Full physical examination (excluding pelvic and rectal examination)

### Vital Signs

Sitting heart rate, SBP and DBP

Temperature

### Laboratory Tests

Urinalysis

ALT

AST

Fasting glucose

2 hour oral glucose tolerance test

HbA1c

IGF-1

### Adverse Events

All adverse events (AEs) and serious AEs (SAEs)

## Assessment Methods

All the assessments, unless otherwise specified, will be performed in the morning (between approximately 6 am and 10 am), before drug intake, on fasting patients (10 to 12-hour fast). Additional time (up to early afternoon) is allowed for the assessments of the Screening and Baseline Visits. Physical examination and height can be assessed at any time during the day regardless of food or drug intake.

### Clinical

#### Physical Examination

A full physical examination (excluding pelvic and rectal examination) including active query, will be performed by the Investigator. Presence or absence of edema at each extremity will be evaluated. Edema will be classified as pitting or non-pitting. Pitting edema is identified by pressing a thumb into the edematous area for approximately 5 seconds and observing that an indentation remains for some time thereafter. Non-pitting edema is identified by pressing a thumb into the edematous area for approximately 5 seconds and observing that no indentation resulted. The severity of edema will be graded on a four-point scale, from slight to very marked.

#### Body Weight

Body weight should be obtained with the subject wearing undergarments or very light clothing and no shoes, and with an empty bladder. The same scale should be used throughout the study. The scale needs to be calibrated before the study is started and its precision should be verified periodically throughout the study and at the completion of the study. The floor surface on which the scale rests must be hard rather than carpeted or covered with other soft material. Self-reported weights are not acceptable. Patients must not read the scale themselves.

#### Blood Pressure

SBP and DBP will be measured using a calibrated automated device. The same model of device will be used for each subject throughout the study. Blood pressure should be measured under standardized conditions, on the same arm. Both SBP and DBP should be recorded. Blood pressure should be measured in sitting position (after the subject has rested comfortably for at least 5 min). At Screening Visit, sitting blood pressure should be checked in both arms on two consecutive readings separated by 2 min. The arm with the higher SBP will be determined at this visit, and blood pressure should be measured using this arm throughout the study.

#### Heart Rate

Heart rate (pulse) will be measured over approximately 30 seconds (by the same automated device used for blood pressure measurement) at the time of the measurement of sitting blood pressure and the results will be recorded.

#### Temperature

Temperature (°C) will be measured at the time of the measurement of sitting blood pressure and the result will be recorded.

#### Resting Energy Expenditure and Respiratory Quotient

Resting energy expenditure and respiratory quotient will be measured using a calibrated automated device. The same model of device will be used for each subject throughout the study. The device should be used according to the manufacturer’s instructions.

#### Electrocardiogram

The electrocardiogram (ECG) will be obtained using a 12-lead cardiograph. ECG will be performed after a 15-min rest in supine position. The ECG results will be assessed by the Investigator.

#### Dual-Energy X-Ray Absorptiometry (DEXA)

The DEXA will be obtained using a Hologic QDR 4500W. Whole body, spine, and hip scans will be performed. The DEXA scan uses a small amount of radiation. Body composition scans include a radiation dose of about 3.3 millirem, while bone density scans include a radiation dose of about 7 millirem. There are no known health effects from this level of radiation exposure. Women of child bearing potential will have a pregnancy test prior to the procedure. No DEXA will be run if the pregnancy test is positive. The DEXA results will be assessed by the Investigator.

#### Two Hour Oral Glucose Tolerance Test

The 2 hour oral glucose tolerance test will be performed after an oral load of 1.75 g/kg to a maximum of 75 g of glucose administered with 240 mL of water. The glucose solution must be consumed within 5 minutes. Blood samples are collected for the measurement of glucose and insulin before and 120 minutes after glucose load.

### Questionnaires

#### Hyperphagia Questionnaire

The hyperphagia questionnaire has been modified from the most recent version of the Dykens questionnaire (9). The modification focuses the questions on the patient’s behavior in the most recent 2 weeks. It consists of 9 multiple choice questions.

#### Behavioral Questionnaire

The behavioral questionnaire consists of 29 questions concerning the presence or absence of behaviors that are characteristic of Prader-Willi syndrome.

#### Adverse Events

All AEs and SAEs will be recorded (see Section 12).

If the presence of any symptoms and/or adverse events is reported while the subject is at the study site, the clinic staff may advise the subject to remain at the site for safety reasons until the staff decides it is safe for the patients to leave.

### Biological

#### Blood Parameters

##### Clinical Laboratory Parameters

Blood samples for clinical laboratory test will be collected by venipuncture and analyzed by the designated laboratory.

The total blood required per subject for clinical laboratory tests will be approximately
70 mL over 126 days.

##### Pharmacokinetic Parameter

Blood samples for pharmacokinetic evaluation will be collected by venipuncture and processed as described below.

##### Sampling Time Points

Blood sampling for the plasma concentration of diazoxide on the visits on Day 69 and Day 97 should take place before drug administration.

The actual time of sample collection should be documented.

##### Pharmacokinetic Sample Volume and Collection Container

The total blood requirement per subject for pharmacokinetic sampling approximately 3 mL

For the sampling, 3 mL of blood will be collected in a K_2_ EDTA vacutainer. The labels for all biological sample collection and storage containers will contain, at a minimum, the subject's number, study number, dosing period, collection date, scheduled collection time (study hour).

##### Pharmacokinetic Sample Collection and Processing

Upon collection, samples will be processed in a timely manner. Samples will be cooled by an ice bath or cooling device, centrifuged at approximately 3000 RPM and 4° C (± 10° C) for 10 minutes, the plasma transferred into duplicate polypropylene tubes, and stored at approximately -20°C (range -10°C to -30°C) until transfer or shipment to the designated bioanalytical laboratory. Approximately 1 mL of plasma will be transferred into the first polypropylene tube (primary) and the remaining plasma will be transferred to the second polypropylene tube (back-up). The time between sample collection and freezer storage should not exceed 1 hour. Each shipment will contain a complete set of samples. The second set of samples will not be shipped until the status of the first shipment is determined.

##### Transport of Pharmacokinetic Samples

The clinical staff will inventory the samples which are to be shipped to the designated bioanalytical laboratory. The inventory record will accompany the frozen plasma samples as per Frontage Laboratories Standard Operating Procedures.

For sample shipment requiring a third party courier, the samples will be packed in ample dry ice within a styrofoam container to ensure the samples will remain frozen for at least 72 hours and shipped via express delivery to the designated bioanalytical laboratory. Written notification of sample shipment will be communicated to the designated bioanalytical laboratory and Sponsor. The samples will be tracked to assure arrival in a safe and timely manner.

The shipment will be accompanied by logs showing the name of the investigational drug product, the protocol number, and the patients and samples included in the shipment. Documentation noting the conditions of the samples upon arrival at the designated bioanalytical laboratory and whether the amount of dry ice remaining is adequate or inadequate will be sent to the clinic.

The samples will be shipped frozen to the designated bioanalytical laboratory whose contact person and address are as follows:

Mira Hong, Ph.D.
Director, Bioanalytical Project Management
Frontage Laboratories, Inc
105 Great Valley Parkway
Malvern, PA 19355
Phone: (484)323-5302

Fax: (610)232-0101

Email: [mhong@frontagelab.com](mailto:mhong@frontagelab.com)

# VISITS

## General Overview

This study involves 8 visits.

The assessments of Visit 2 (Baseline Visit) through the last visit, Visit 8, should take place as scheduled; a ± 2 day visit window is allowed.

## Assessments

The following procedures will be performed (Also see Section 10 Study Endpoints).

### Visit 1 (Day -28 to -14; Screening)

During the 42-day Screening Period, the Screening procedures must be done within the interval from Day -42 to Day -28 and can be all done on one day or spread over multiple days. Thus all screening procedures must be completed by Day -28.

Informed consent

Demographic characteristics (gender, age, ethnic origin)

Medical history

Physical examination

Height

Weight

Waist circumference

Vital signs (sitting heart rate and blood pressure, and temperature)

Hyperphagia questionnaire

ECG

Fasting Glucose

HbA1c

AST

ALT

IGF-1

Urinalysis

Previous (within 1 month) and concomitant medication record

AEs record

### Visit 2 (Day 0; Baseline; Start of Treatment)

Vital signs (sitting heart rate and blood pressure, and temperature)

Physical exam

Weight

Waist circumference

2 hour oral glucose tolerance test

Ghrelin

Leptin

Resting energy expenditure

Respiratory quotient

Urine (dipstick) pregnancy test in women of child-bearing age must be administered and read before DEXA

DEXA

Hyperphagia questionnaire

Behavioral questionnaire

Fasting triglyceride

Fasting total cholesterol

Fasting LDL cholesterol (calculated)

Fasting HDL cholesterol

Fasting non-HDL cholesterol (calculated)

Comprehensive metabolic panel

Enrollment

Concomitant medication record

Accountability and dispensation of investigational drug

Dose administration in clinic, within 15 min of food consumption that includes solid food.

AEs record

(Note: Patients will need to bring their investigational drug product to each visit.)

### Visit 3 (Day 13)

Physical examination

Weight

Waist circumference

Fasting glucose

Vital signs (sitting heart rate and blood pressure, and temperature)

Hyperphagia questionnaire

Concomitant medication record

Decision about dose titration or adjustment

Accountability and dispensation of investigational drug product

Dose administration in clinic, within 15 min of food consumption that includes solid food.

AEs record

### Visit 4 (Day 27)

Physical examination

Weight

Waist circumference

Fasting glucose

Resting energy expenditure

Respiratory quotient

Hyperphagia questionnaire

Vital signs (sitting heart rate and blood pressure, and temperature)

Concomitant medication record

- Decision about dose titration or adjustment

Accountability and dispensation of investigational drug product

Dose administration in clinic, within 15 min of food consumption that includes solid food.

AEs record

### Visit 5 (Day 41)

Physical examination

Weight

Waist circumference

Fasting glucose

Vital signs (sitting heart rate and blood pressure, and temperature)

Hyperphagia questionnaire

Concomitant medication record

- Decision about dose titration or adjustment

Accountability and dispensation of investigational drug product

Dose administration in clinic, within 15 min of food consumption that includes solid food.

AEs record

### Visit 6 (Day 55)

Physical examination

Weight

Waist circumference

Fasting Glucose

Vital signs (sitting heart rate and blood pressure, and temperature)

Concomitant medication record

Decision about dose titration or adjustment

Accountability and dispensation of investigational drug product

Dose administration in clinic, within 15 min of food consumption that includes solid food.

AEs record

### Visit 7 (Day 69; End of the Open Label Treatment Period)

Vital signs (sitting heart rate and blood pressure, and temperature)

Physical exam

Weight

Waist circumference

HbA1c

2 hour oral glucose tolerance test

Ghrelin

Leptin

Resting energy expenditure

Respiratory quotient

Urine (dipstick) pregnancy test in women of child-bearing age must be administered and read before DEXA

DEXA

Hyperphagia questionnaire

Behavioral questionnaire

Fasting triglyceride

Fasting total cholesterol

Fasting LDL cholesterol (calculated)

Fasting HDL cholesterol

Fasting non-HDL cholesterol (calculated)

Comprehensive metabolic panel

Urinalysis

AST

ALT

IGF-1

Pharmacokinetic sampling

Concomitant medication record

Accountability investigational drug product

Dose administration in clinic, within 15 min of food consumption that includes solid food.

AEs record

Randomization in the Double-Blind, Placebo-Controlled, Randomized Withdrawal Extension

Dispensation of investigational drug product

### Visit 8 (Day 97; End of Study)

Vital signs (sitting heart rate and blood pressure, and temperature)

Physical exam

Weight

Waist circumference

HbA1c

2 hour oral glucose tolerance test

Ghrelin

Leptin

Resting energy expenditure

Respiratory quotient

Hyperphagia questionnaire

Fasting triglyceride

Fasting total cholesterol

Fasting LDL cholesterol (calculated)

Fasting HDL cholesterol

Fasting non-HDL cholesterol (calculated)

Comprehensive metabolic panel

Urinalysis

AST

ALT

IGF-1

Pharmacokinetic sampling

Concomitant medication record

Accountability of investigational drug

AEs record

# SAFETY MONITORING AND REPORTING

## Adverse Events

### General Definitions

#### Adverse Event (AE)

An AE is any untoward medical occurrence in a subject administered a pharmaceutical product.

#### Unexpected AE

An unexpected AE is an untoward medical occurrence not previously reported or an AE that occurs with specificity, severity, or frequency that is not consistent with the current Clinical Investigator’s Brochure.

### Causality Assessment of AE

All AEs regardless of seriousness or relationship to the investigational drug product including those occurring during the screening period (after the signing of the Informed Consent Form) are to be recorded in the appropriate CRF pages. The Investigator should specify the date of onset, severity (mild, moderate, severe), action taken with respect to the investigational drug product, corrective treatment, outcome, and whether or not there is a reasonable possibility that the AE may have been caused by the investigational drug product. All AEs that occur during the period between Screening and dose administration at the Baseline visit should be clearly identified as being unrelated to study medication.

The assessment of the relationship of an AE to the administration of the investigational drug product (yes, no) is a clinical decision based on all available information at the time of the completion of the CRF:

Yes: the time course between the administration of the investigational drug product and the occurrence or worsening of the AE is consistent with a causal relationship and no other cause can be identified (concomitant drugs, therapies, complications, etc.).

No: the time course between the administration of the investigational drug product and the occurrence or worsening of the AE rules out a causal relationship and another cause (concomitant drugs, therapies complications, etc.) is suspected.

### Abnormalities of Clinical and Laboratory Assessments

Abnormalities of vital signs, and laboratory results are to be recorded as AEs only if they are considered by the Investigator as clinically significant (symptomatic, requiring corrective treatment, leading to discontinuation, fulfilling a seriousness criterion).

### Treatment Discontinuation and Follow-Up

In the case of a treatment discontinuation due to a non-serious adverse event the Investigator should immediately fill-in, sign and date the corresponding page(s) in the CRF.

The Investigator should follow-up the outcome of any AE (clinical signs, laboratory values, etc.) until the return to normal or consolidation of the subject’s condition. If the AE is present more than 30 days after the last dose of the investigational drug product, the Investigator should consider the need for the continuation of the follow-up.

### Other Considerations

Any pre-existing conditions or signs and/or symptoms present in a subject prior to the Screening Visit should be recorded as medical/surgical history.

## Serious Adverse Events

### Definition of Serious Adverse Event (SAE)

A SAE is any untoward medical occurrence that:

is a congenital anomaly/birth defect, or

is a medically important event, or

is life-threatening, or

requires hospitalization or prolongation of hospitalization (elective hospitalizations and/or hospitalizations for treatment of pre-existing conditions that did not worsen from Baseline are not considered AEs and should not be reported as SAEs), or

results in persistent or significant disability, or

results in death

### SAE Communication and Follow-Up

All SAEs must be communicated to the sponsor immediately. The sponsor is responsible for communicating SAEs to the FDA when required. The Investigator must follow-up the outcome of any SAE (clinical signs, laboratory values, etc.) until the return to normal or consolidation of the subject’s condition.

## Safety Instructions Specific to the Study

### Hyperglycemia

A mild elevation of fasting glucose may be observed during the first weeks of treatment with diazoxide choline, which are mostly transient and self-limiting. Rarely, more pronounced hyperglycemia may develop, regular monitoring is recommended. Hyperglycemia resolves rapidly with treatment cessation.

### Fluid Retention and Peripheral Edema

The fluid retention and peripheral edema associated with diazoxide choline administration, appears to resolve or improve while treatment continues, and will resolve rapidly once treatment ends. Persistent or marked edema responds rapidly to diuretic or drug holiday. It should be noted that peripheral edema associated with diazoxide choline treatment is not likely to be associated with congestive heart failure in the study population.

### Electrocardiographic Changes

Electrocardiographic ST-T wave changes (mostly T wave flattening, occasionally T wave inversion, and rarely minimal ST depression) may be observed during treatment with diazoxide choline. These changes bear no relationship to myocardial ischemia as detected with stress echocardiography or other cardiovascular adverse effects, are considered to be clinically benign, and revert to normal while treatment continues or after treatment discontinuation. If new ST-T wave changes of this sort occur concomitantly with chest pain or other symptoms or findings that are consistent with myocardial ischemia, treatment should be discontinued and prompt medical evaluation (including cardiology consultation if necessary) should be obtained. If such changes occur without symptoms or other findings suggesting myocardial ischemia, no further investigation is required.

### Elevation of Serum Uric Acid

Diazoxide may increase the level of serum uric acid and this should be monitored in patients with hyperuricemia or a history of gout.

### Consideration in Use with Concomitant Antihypertensive Agents

The antihypertensive effect of DCCR is a direct pharmacological action of the drug and should be kept in mind when administering DCCR concomitantly with other antihypertensive agents.

### Effects on Anticoagulants

Diazoxide is highly bound to serum protein. It may displace other substances which are also bound to protein, such as coumarin and its derivatives; resulting in higher blood levels of these substances. Because of protein binding, administration of DCCR with coumarin or its derivatives may require reduction in the dosage of the anticoagulant. There has been no reported evidence of excessive anticoagulant effect.

# TREATMENT OR STUDY DISCONTINUATION

## Treatment Discontinuation

### Reasons

The subject may withdraw from the treatment with the investigational drug product at any time and irrespective of the reason.

The investigator may decide to discontinue the investigational drug product for the following reasons:

Poor subject compliance and/or major protocol deviation

Adverse events

Significant intolerance to the investigational drug product

### Procedures

When confirmed, a permanent treatment discontinuation should be recorded by the Investigator in the appropriate CRF pages.

If the discontinuation occurs during the Open label Treatment Period, every effort should be made to complete the clinical and laboratory evaluations scheduled for the last visit of the Open Label Treatment Period, i.e. Visit 7.

If the discontinuation occurs during the Double-Blind, Placebo-Controlled, Randomized Withdrawal Extension, every effort should be made to complete the clinical and laboratory evaluations scheduled for the only visit of the Extension, i.e. Visit 8.

## Study Discontinuation

### Reasons

The subject may withdraw from the study at any time and irrespective of the reason.

The Investigator may decide to withdraw the subject from the study if this is considered to be in the best interest of the subject.

### Procedures

When confirmed, a study discontinuation should be recorded by the Investigator in the appropriate CRF pages.

If the discontinuation occurs during the Open label Treatment Period, every effort should be made to complete the clinical and laboratory evaluations scheduled for the last visit of the Open Label Treatment Period, i.e. Visit 7.

If the discontinuation occurs during the Double-Blind, Placebo-Controlled, Randomized Withdrawal Extension, every effort should be made to complete the clinical and laboratory evaluations scheduled for the only visit of the Extension, i.e. Visit 8.

Patients who have withdrawn from the study cannot be re-enrolled in the study. Their screening number and subject number must not be re-used. Patients who discontinued the study will not be replaced.

# STATISTICAL ANALYSIS

The following describes the statistical methods to be used in the analysis of the data.

## Sample Size

A sample size of up to 12 patients is planned for the study. It is based on empirical consideration.

## Analysis Populations

### Open Label Treatment Period Endpoint Evaluable Treatment Population

Patients who have sufficient data from Day 13 (the first post-Baseline visit) to Day 69 will be included in the Open Label Treatment Period endpoint evaluable population.

### Double-Blind, Placebo-Controlled, Randomized Withdrawal Extension Endpoint Evaluable Population

Patients who were randomized in the Double-Blind, Placebo-Controlled, Randomized Withdrawal Extension and have sufficient data from Day 70 to Day 97 will be included in the Double-Blind, Placebo-Controlled, Randomized Withdrawal Extension endpoint evaluable population.

### Safety Population

Patients who take at least 1 dose of investigational drug product will be assessed for safety.

## Sample Size and Power

### Statistical Hypotheses and Control of the False Positive Rate

For the period from Baseline to Day 69, the null hypotheses for the three endpoints—weight, resting energy expenditure (as % RDA) and RQ—are that the mean changes in the parameters are zero. The alternative hypotheses are that the endpoints mean changes are those in Table 1. For the period from Day 69 to Day 97, the null hypotheses are that the two treatment arms have the same mean changes between Days 69 and 97. The alternative hypotheses are that the mean change between Day 69 and Day 97 is zero in the treated group and that the means return to baseline levels in the control group.

Given the exploratory nature of the study, power was evaluated at α values of 0.05, 0.10 and 0.15, ignoring the multiplicity of endpoints.

### Assumptions for Sample Size and Power

The assumptions for alternative hypotheses were derived from unpublished results from one of the sponsor’s studies, from published results and from statistical derivations.

Weight change from baseline or percent change from baseline was obtained from two clinical studies in which diazoxide or DCCR were titrated through a similar dose range in obese patients. In the clinical study by van Boekel (6) diazoxide treatment was initiated in 18 obese male subjects at a dose of 300 mg/day and titrated at one month to 450 mg/day. Weight and standard deviation of weight were presented on a monthly basis. Data from the two month (8.66 weeks) timepoint was used. In Essentialis clinical study TR002, DCCR treatment was initiated at 217.5 mg/day in 11 obese subjects and titrated in weekly steps to a dose of 507.5 mg/day. The study continued for 8 weeks. Percent weight loss was recorded by subject. Slight greater weight loss was observed in clinical study TR002 at 8 weeks than was observed in the study reported by van Boekel through 2 months of treatment. The anticipated weight loss in this power analysis was taken as a weight average of the two clinical studies, while the estimate of standard deviation of percent change came from clinical study TR002 where individual subject data was available.

Baseline estimates of resting energy expenditure, expressed as % RDA and RQ and the variability in the baseline measurements were obtained from a publication on nutritional phases of PWS by Miller et al. (8). The estimated change due to treatment for both of these variables comes from unpublished data on a small number of PWS patients treated with the currently approved diazoxide product. The changes observed in these patients were very consistent with the changes in these variables observed in relevant animal models.

The estimation of standard deviation of a change is based on the standard deviations of the pre and post measurements and the correlation between subjects’ pre and post measurements, which was assumed to be 0.5. The calculated values of means and standard deviation on differences are shown in bold italics in Tables 1 and 2.

Additional assumptions were the use of two-sided statistical tests and Gaussian distributions.

### Power for the period from Baseline to Day 69

The power calculations for change from Baseline to Day 69 were based on a sample size of 12 subjects. Assumptions on alternative hypotheses for the endpoints and the resulting statistical power are presented in Table 1.

**Table 1. Power calculations for weight, resting energy expenditure and respiratory quotient for the period Baseline to Day 69**

|  |  | Endpoint | | |
| --- | --- | --- | --- | --- |
|  |  | Weight change (%) | Resting energy expenditure  (% RDA) | Respiratory quotient (RQ) |
| Assumption: Mean | Pre | n/a | 44.0 | 0.86 |
|  | Post | n/a | 74.0 | 0.75 |
|  | Change | 4.9 | ***30.0*** | ***-0.11*** |
| Assumption: Standard Deviation | Pre | n/a | 13.6 | 0.12 |
|  | Post | n/a | 13.6 | 0.12 |
|  | Change | 3.7 | ***13.6*** | ***0.12*** |
| Result: Statistical power (%) | α = 0.05 | 98.6 | >99.9 | 82.4 |
|  | α = 0.10 | 99.6 | >99.9 | 90.8 |
|  | α = 0.15 | 99.8 | >99.9 | 94.3 |

### Power for the period from Day 69 to Day 97

The power calculations for the period from Day 69 to Day 97 are based sample sizes of 6 subjects per group. Assumptions on alternative hypotheses for the endpoints and the resulting statistical power are presented in Table 2.

**Table 2. Power calculations for weight, resting energy expenditure and respiratory quotient for the period Day 69 to Day 97**

|  |  | Endpoint | | |
| --- | --- | --- | --- | --- |
|  |  | Weight change (%) | Resting energy expenditure  (% RDA) | Respiratory quotient |
| Assumption: Mean | Pre | n/a | 44.0 | 0.86 |
|  | Post | n/a | 74.0 | 0.75 |
|  | Change | 4.9 | ***30.0*** | ***-0.11*** |
| Assumption: Standard Deviation | Pre | n/a | 13.6 | 0.12 |
|  | Post | n/a | 13.6 | 0.12 |
|  | Change | 3.7 | ***13.6*** | ***0.12*** |
| Result: Statistical power (%) | α = 0.05 | 54.4 | 93.0 | 30.1 |
|  | α = 0.10 | 68.8 | 97.1 | 43.5 |
|  | α = 0.15 | 76.8 | 98.5 | 52.7 |

## Statistical Methods

### Descriptive Analysis

Descriptive statistics (number of observations, mean, standard deviation, median, minimum and maximum) will be reported for all secondary and exploratory endpoints for the Open Label Treatment Period endpoint evaluable population for endpoints measured during the Open Label Treatment Period. These descriptive statistics will be reported by treatment arm for Double-Blind, Placebo-Controlled, Randomized Withdrawal Extension endpoint evaluable population and by treatment arm for the responder sub-group for all secondary and exploratory endpoints measured during the Double-Blind, Placebo-Controlled, Randomized Withdrawal Extension. Baseline for hyperphagia, will be defined as the average of measurements made at the Screening and Baseline visits.

Graphs may be created to present a visual assessment of the relationship between dose titration and response on hyperphagia.

### Paired T-Tests

All exploratory endpoints measured during the Open Label Treatment Period including hyperphagia, resting energy expenditure (expressed as % RDA), RQ, BMI, waist circumference, body fat mass, ghrelin and leptin change from Baseline through Day 69, and weight, fasting triglyceride, fasting total cholesterol, fasting LDL cholesterol, fasting HDL cholesterol, fasting non-HDL cholesterol percent change from Baseline through Day 69 will be analyzed by paired t-tests.

### ANOVA

An analysis of variance will be conducted on the secondary endpoints measured during the Double-Blind, Placebo-Controlled, Randomized Withdrawal Extension, including hyperphagia and resting energy expenditure (expressed as % RDA) change from Day 69 to Day 97, comparing the treatment arm with the placebo arm.

An analysis of variance will be conducted on the exploratory endpoints measured during the Double-Blind, Placebo-Controlled, Randomized Withdrawal Extension, including RQ, BMI, waist circumference, ghrelin and leptin change from Day 69 through Day 97, and weight, fasting triglyceride, fasting total cholesterol, fasting LDL cholesterol, fasting HDL cholesterol, fasting non-HDL cholesterol percent change from Day 69 through Day 97, comparing the treatment arm with the placebo arm.

Similar analysis will also be conducted on the responder subgroup.

### Fisher’s Exact Test

Adverse events that are newly arisen in the Double-Blind, Placebo-Controlled, Randomized Withdrawal Extension will be summarized by treatment arm and analyzed by Fisher’s exact test.

## Demographic and Baseline Characteristics

Demographic and Baseline characteristics will be described.

### Study Medication

#### Compliance

Compliance with taking of the investigational drug product will be summarized.

### Concomitant Medications

Concomitant medications will be listed for each subject. Selected medications or classes of medications may be summarized.

## Study Endpoints

### Safety Endpoints

The following safety characteristics will be summarized.

#### Physical Examination

Parameters include incidence of clinically significant abnormal findings as well as incidence and severity of peripheral edema.

#### Vital Signs

Parameters include heart rate, blood pressure, and temperature.

#### Laboratory Tests

Parameters include numeric values of laboratory parameters and their changes from Baseline value.

#### DEXA Scan

Parameters include numeric values of total body fat (identified as an exploratory endpoint) and changes from Baseline value.

#### Adverse Events

AEs will be analyzed for incidence, severity, and attribution for the entire population for the period from Baseline through Day 69 and by treatment group for the period from Day 69 through Day 97. SAEs will be similarly analyzed.

## Secondary Efficacy Endpoints

- Hyperphagia change from Day 69 through Day 97 using hyperphagia questionnaire
- Resting energy expenditure expressed as percent RDA change from Day 69 through Day 97

## Exploratory Efficacy Endpoints

- Hyperphagia change from Baseline through Day 69 using hyperphagia questionnaire
- Resting energy expenditure expressed as percent RDA change from Baseline through Day 69
- Weight change from Baseline through Day 69
- Waist circumference change from Baseline through Day 69
- BMI change from Baseline through Day 69
- Body fat mass (as measured by DEXA) change from Baseline through Day 69
- Respiratory quotient at Baseline and Day 69
- Ghrelin change from Baseline through Day 69
- Leptin change from Baseline through Day 69
- Triglyceride percent change from Baseline through Day 69
- Total cholesterol percent change from Baseline through Day 69
- LDL cholesterol percent change from Baseline through Day 69
- HDL cholesterol percent change from Baseline through Day 69
- Non-HDL cholesterol percent change from Baseline through Day 69
- Weight change from Day 69 through Day 97
- Waist circumference change from Day 69 through Day 97
- BMI change from Day 69 through Day 97
- Respiratory quotient change from Day 69 through Day 97
- Ghrelin change from Day 69 through Day 97
- Leptin change from Day 69 through Day 97
- Triglyceride percent change from Day 69 through Day 97
- Total cholesterol percent change from Day 69 through Day 97
- LDL cholesterol percent change from Day 69 through Day 97
- HDL cholesterol percent change from Day 69 through Day 97
- Non-HDL cholesterol percent change from Day 69 through Day 97

# STUDY COMMITTEES

## Steering Committee

No Steering Committee is planned for this study.

## Data and Safety Monitoring Board

A Data and Safety Monitoring Board is planned for this study. Membership will consist of medical staff of the University of California, Irvine School of Medicine, and includes Dr. Maria Cristina Kenney, Professor, Dr. John Jay Gargus, Professor, and Dr. Madeleine Pahl, Professor.

# ETHICAL CONSIDERATIONS

## Principles

The study will be conducted in accordance with the principles established by the 18th World Medical Assembly (Helsinki, 1964) and all applicable amendments established by the World Medical Assemblies and the ICH guidelines for Good Clinical Practices (GCP).

## Informed Consent

The Investigator (or a designated representative) should fully inform the subject of all relevant aspects of the study. Prior to the study start, the written Informed Consent Form must be dated and signed by the subject and by the person who conducted the informed consent discussion. A copy of the dated and signed Informed Consent Form will be provided to the subject.

## Ethics Committee

The Investigator must submit this Clinical Trial Protocol (together with the Informed Consent Form, the Investigator’s Brochure, the Investigator’s curriculum vitae, and any other relevant document) to the appropriate Ethics Committee (Institutional Review Board, “IRB”), and is required to forward to the Sponsor a copy of the written and dated favorable opinion signed by the Chairman of the Ethics Committee with information on the composition of the Committee. The investigational drug product will not be released at the study site and the study will not start until this copy has been received by the Sponsor.

During the course of the study, any amendment to the Clinical Trial Protocol or any update to the Investigator’s Brochure will be sent to the Ethics Committee.

If requested, a summary of the study outcome will be sent to the Ethics Committee at the end of the trial.

## Confidentiality

All information produced during the study (Clinical Trial Protocol Amendment, study results, etc.) is confidential. The Investigator and the Sub-Investigators agree to keep this information confidential and not to disclose it to any third party without the prior written approval of the Essentialis. The Ethics Committee members have also the same obligation of confidentiality.

# ADMINISTRATIVE AND REGULATORY PROCEDURES

## Laws

The study will be conducted in compliance with US laws, regulations, and guidelines.

## Clinical Trials Registration

This trial will be registered in appropriate clinical trial database(s) consistent with federal law and regulation, FDA guidance, and WHO mandate.

## Curriculum Vitae

An updated copy of the curriculum vitae of the Investigator and the Sub-Investigators will be provided to the Sponsor prior to the start of the study.

## Record Retention

The Investigator must maintain confidential all study documentation and prevent accidental or premature destruction of the documents. The documents should be retained at least 15 years after the completion of the study. If archiving can no longer be ensured by the Investigator, the Sponsor should be informed and a mutually agreed upon alternative should be proposed. The Investigator must notify the Sponsor prior to destroying any document before the 15-year period.

## Data Protection

The Sponsor should treat the subject’s personal data and the Investigator’s personal data in compliance with all applicable laws and regulations, and take all appropriate measures to protect the data and prevent access to the data by an unauthorized third party.

## Insurance

The Investigator certifies that have a liability insurance policy covering the liability of the Investigator and Sub-Investigators. The insurance policy is in accordance with local laws and requirements.

# CLINICAL TRIAL PROTOCOL AMENDMENTS

Any amendment to the Clinical Trial Protocol requires a written favorable opinion from the Ethics Committee prior to its implementation, unless there are overriding safety reasons. In case a change to the Informed Consent Form becomes necessary following the amendment, the Investigator must receive a favorable opinion from the Ethics Committee prior to the implementation of the change.

# STUDY MONITORING

## Responsibilities of the Investigator

The Investigator is responsible to conduct the study in accordance with the Clinical Trial Protocol, the ICH guidelines for GCP, and the applicable regulatory requirements. The Sub-Investigators appointed to assist the Investigator will be under direct responsibility of the Investigator. The Investigator agrees to provide reliable data as requested by the Clinical Trial Protocol (through CRF and discrepancy resolution form, “DRF”) in an accurate and legible manner and to ensure direct access to source documents to the Sponsor’s representatives.

# Premature Discontinuation of the Study

## Decision by the Investigator

The Investigator may decide to discontinue the study or to close the site for the following reasons:

- The Investigator has information on the investigational drug product leading to a doubt about the benefit/risk ratio
- The Investigator must notify the Sponsor (prior notice of 30 days) of this decision and give the reason in writing. The Ethics Committee and Health Authorities should also be informed.

# PROPERTY RIGHTS -wording

All information, documents, and investigational drug product provided by the Sponsor remain the property of the Sponsor. All the results arising from the study remain the property of the Sponsor.

# Communication and Publication of study Results

The Investigator has the right to publish the results of the study. The Investigator has the right to communicate and publish the results of the study after obtaining written consent from the company having rights to DCCR.

# Inspections and Audits

To ensure compliance with the Clinical Trial Protocol, the GCP, and the applicable regulatory requirements, the Investigator may be audited by the Sponsor or by the regulatory authorities (FDA). The Investigator agrees to allow the auditors to have direct access to the study records for review. The confidentiality of the data audited should be respected by all parties. Corrective actions should be implemented for all problems found during the audit.

# References

1. Diazoxide Choline Clinical Investigator’s Brochure. Version 2, March 21, 2009
2. Pruitt AW, et al. Disposition of diazoxide in children. Clin Pharmacol Ther, 1973, 14(1): 73-82
3. Sellers E and Koch-Weser J. Protein binding and vascular activity of diazoxide. New Eng J Med, 1969, 281(21): 1141-1145
4. Pruitt AW, et al. Metabolism of diazoxide in man and experimental animals. J Pharmacol Exp Therap 1974 188(1): 248-256
5. Alemzadeh, R, et al. Beneficial effects of diazoxide in obese hyperinsulinemic adults. J Clin Endocrinol Metab 1998; 83:1911–1915.
6. Van Boekel, G, et al. Weight loss in obese men by caloric restriction and high-dose diazoxide–mediated insulin suppression. Diabetes Obes Metab 2008; 10(12):1195-1203.
7. Hamilton, JK, et al. Hypothalamic obesity following craniopharyngioma surgery: Results of a pilot trial of combined diazoxide and metformin therapy. Inter J Pediatric Endocrin 2011:417949. Epub.
8. Miller, JL, et al. Nutritional phases in Prader-Willi syndrome. Am J Med Genet A 2011 155A(5):1040-1049
9. Dykens, EM, et al. Assessment of hyperphagia in Prader-Willi syndrome. Obesity 2007 15(7):1816-1826

# Appendices

## Laboratory for Pharmacokinetic Measurements

Mira Hong, Ph.D.

Frontage Laboratories, Inc.

105 Great Valley Parkway

Malvern, PA 19355

Phone: (484) 323-5302

Fax: (610) 232-0101
